# Annexin Animal Models—From Fundamental Principles to Translational Research

**DOI:** 10.3390/ijms22073439

**Published:** 2021-03-26

**Authors:** Thomas Grewal, Carles Rentero, Carlos Enrich, Mohamed Wahba, Carsten A. Raabe, Ursula Rescher

**Affiliations:** 1 School of Pharmacy, Faculty of Medicine and Health, University of Sydney, Sydney, NSW 2006, Australia; mwah4790@uni.sydney.edu.au; 2 Departament de Biomedicina, Unitat de Biologia Cel·lular, Facultat de Medicina i Ciències de la Salut, Universitat de Barcelona, 08036 Barcelona, Spain; carles.rentero@ub.edu (C.R.); enrich@ub.edu (C.E.); 3 Centre de Recerca Biomèdica CELLEX, Institut d’Investigacions Biomèdiques August Pi i Sunyer (IDIBAPS), 08036 Barcelona, Spain; 4 Research Group Regulatory Mechanisms of Inflammation, Center for Molecular Biology of Inflammation (ZMBE) and Cells in Motion Interfaculty Center (CiM), Institute of Medical Biochemistry, University of Muenster, 48149 Muenster, Germany; raabec@uni-muenster.de

**Keywords:** annexins, calcium, human disease models, KO mice, membrane trafficking, membrane organization, extracellular annexin functions

## Abstract

Routine manipulation of the mouse genome has become a landmark in biomedical research. Traits that are only associated with advanced developmental stages can now be investigated within a living organism, and the in vivo analysis of corresponding phenotypes and functions advances the translation into the clinical setting. The annexins, a family of closely related calcium (Ca^2+^)- and lipid-binding proteins, are found at various intra- and extracellular locations, and interact with a broad range of membrane lipids and proteins. Their impacts on cellular functions has been extensively assessed in vitro, yet annexin-deficient mouse models generally develop normally and do not display obvious phenotypes. Only in recent years, studies examining genetically modified annexin mouse models which were exposed to stress conditions mimicking human disease often revealed striking phenotypes. This review is the first comprehensive overview of annexin-related research using animal models and their exciting future use for relevant issues in biology and experimental medicine.

## 1. Introduction

The assembly of the human genome, followed by DNA deep sequencing, changed our approach to analyse gene functions. Genetic tools, which helped to develop a major part of our understanding of modern biology, started off with phenotypes and aimed to identify the underlying gene or its allelic variation to infer function. The completion of the human genome assembly marked the onset of methods that build on reverse genetics. Genes and their genomic sequence became available and the generation of mouse models with gene deletions or targeted mutations allowed the in vivo analysis of corresponding phenotypes and functions.

Mouse models lacking individual members of the annexin gene family have been established and unravelled some of their in vivo functions. There are many excellent reviews on the annexin family covering the common and specific characteristics of individual annexin family members [1,2,3,4,5,6,7,8,9,10,11,12,13]. Hence, in the following, we provide only a brief overview necessary to appreciate the generation and functional implications from the in vivo analysis of the annexin mouse models.

**Figure 1:**
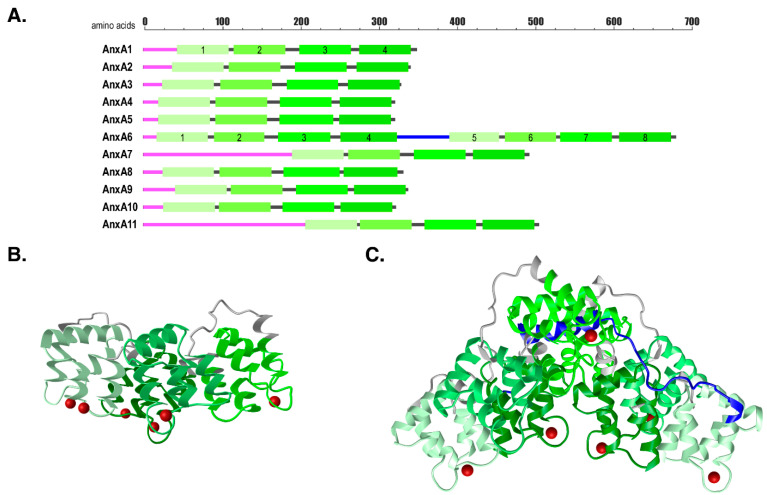
Annexin structural organization. (**A**) Schematic overview of annexins. Magenta, N-terminal tails; light to dark green, C-terminal core domains with Annexin repeats 1–4 and 5–8 for AnxA6; grey, short spacer regions between tail and first Annexin repeat or between Annexin repeats; blue, AnxA6 linker region. (**B**) 3D-structures of human AnxA1 (PDB: 1AIN [17]) and (**C**) bovine AnxA6 (PDB: 1AVC [18]) cores (light to dark green, spacer regions in grey, AnxA6 linker region in blue), with coordinated calcium ions (red). 3D-structures are visualized with the iCn3D software vs. 2.24.6; (https://www.ncbi.nlm.nih.gov/Structure/icn3d/icn3d.html; accessed 12 March 2021).

The annexin family consists of twelve evolutionary conserved and structurally related Ca^2+^- and phospholipid-binding proteins [1,14]. All annexins are structurally related, they encode a variable N-terminal domain, which is responsible for different, annexin member-specific functions, and a highly conserved C-terminal core. The C-terminal domain is composed of four highly related repeats (eight in case of AnxA6) containing Ca^2+^ binding sites that enable the interaction with negatively charged phospholipids (Figure 1). Extensive in vitro analysis solved the three-dimensional structures of annexins ([15,16,17,18], reviewed in [2,19,20]), their Ca^2+^-dependent affinities to various phospholipids and other interaction partners [1,2]. A large number of regulatory roles associated with individual annexins can be explained by their Ca^2+^-inducible and rapid translocation from the cytosol to the plasma membrane or intracellular membranes [1,2,3,4]. At these locations, their unique N-termini interact with dissimilar binding partners, which in part explains the functional diversity of annexins. In addition, the composition and distribution of phospholipids and other lipids, such as phosphatidylinositol-4,5-bisphosphate, cholesterol and ceramide within specific membrane microdomains, but also posttranslational modifications and pH, contribute to different spatiotemporal and Ca^2+^-sensitive membrane binding kinetics leading to further functional diversification of individual family members [1,2,3,4,5,6,7,8,9,11].

These processes are thought to control basic cellular functions that are pivotal to the regulation of growth, development, programmed cell death, but also cell motility, membrane repair and inflammatory response [1,2,3,4,5,6,7,8,9,10,11,12,13]. Ectopic overexpression or siRNA-mediated knockdown delivered great insights in the annexin biochemistry and broadened our knowledge substantially. More advanced technologies of reverse genetics made knockout (KO) mouse models the gold standard to analyse annexin functions. During the last two decades KO-mice strains with targeted deletion of several annexins (i.e., AnxA1, A2, A4, A5, A6, A7 and A8) have been successfully generated and provide an important research tool to the community (Figure 1). All but one of these mice strains did not display any obvious phenotype and developed entirely normal. Given the high degree of conservation this outcome was not anticipated and suggested that potentially individual members of the annexin gene family could functionally substitute for each other and hence might be redundant. However, annexin KO-mice or wildtype mice with implanted annexin KO-cells developed strong phenotypes when analysed in stress conditions that mimicked certain disease [10]. Additionally, the observed phenotypes were specific to individual annexins; a finding which strongly argued against functional redundancy of individual gene family members. In fact, as outlined further below, annexins in general might provide a toolbox to cope with cell stress and pathogen attack [12,13]. Of late, a substantial number of new studies exploring human disorders in annexin KO-models further emphasize their great therapeutic value and the suitability of whole-body annexin KO-mice or animals implanted with annexin-deficient cell lines to investigate a wide range of human diseases.

The following sections address individual annexins and their corresponding functions in rodent and other animal models in vivo.

## 2. AnxA1

AnxA1 is expressed in most cells and tissues, being highly prominent in macrophages, neutrophils, the nervous and endocrine system [1,2,10,21,22]. Inside cells, AnxA1 is located at the plasma membrane, endosomal and secretory vesicles, but also at cytoskeletal elements and in the nucleus. At these sites, AnxA1 contributes to the regulation of endo- and exocytosis, signal transduction, cellular metabolism, and cytoskeletal rearrangements, all relevant for proliferation, differentiation, migration, survival, but also repair, inflammation and viral infection [1,2,3,5,6,11]. In addition, upon glucocorticoid stimulation, AnxA1 is secreted from cells, possibly via non-conventional pathways [1,21,22], delivering substantial amounts of AnxA1 to the extracellular space that drive the multiple anti-inflammatory aspects of glucocorticoid action [1,21,22,23].

### 2.1. AnxA1 and the Anti-Inflammatory Response

An extensive body of work has linked AnxA1 with the regulation of inflammatory processes. Examining glucocorticoid-dependent activation of innate immune cells in the AnxA1 KO-mice (*Anxa1*^−/−^) provided the first in vivo evidence that AnxA1 limits pro-inflammatory response [24] (Table 1a). Complementing earlier in vivo studies administering recombinant AnxA1 [23,25], *Anxa1*^−/−^ mice displayed a boosted immune response characterized by an increased leucocyte migratory behaviour and a substantial resistance to the anti-inflammatory action of glucocorticoids in several acute inflammation models [24]. Mechanistically, in carrageenan-induced paw oedema and zymosan-induced peritonitis, the prominent ability of AnxA1 to counteract inflammatory events occured through binding of extracellular AnxA1 to formyl peptide receptors (FPRs), a family of G-protein coupled receptors found on many cells of the innate immune system, including neutrophils and monocytes [26,27]. The AnxA1/FPR2 axis inhibits neutrophil recruitment to sites of inflammation, a critical step in the regulation of inflammation [28]. In addition, AnxA1 increases neutrophil apoptosis and recruitment of monocytes, which upon differentiation [29], allow clearance of apoptotic cells. The latter might be mediated by locally produced steroids, including estrogens, which promote production and release of AnxA1 [30]. Furthermore, the ability of AnxA1 to reduce production of pro-inflammatory mediators, such as eicosanoids, nitric oxide, and interleukins (ILs) also contributes to resolve inflammation.

The AnxA1/FPR2 axis is now well believed to elicit anti-inflammatory activity through multiple signaling modules, including mitogen-activated protein kinases (MAPK) such as extracellular signal-regulated kinases 1/2 and p38MAPK, as well as protein kinase B (Akt), c-Jun N-terminal kinase, and intracellular Ca^2+^ elevation. Several chemokine receptors [31,32] and transcription factors also act downstream the AnxA1/FPR2 pathway [22,33]. Given the enormous variety of experimental mouse models to study inflammation, we recommend excellent reviews [21,22,23,25,33,34,35,36] for a more comprehensive list of studies that utilized the AnxA1^−/−^ strain to corroborate the anti-inflammatory roles of AnxA1 (Table 1a,b).

Several recent studies have provided additional mechanistic insights. Mast cells critically mediate early lipopolysaccharide-induced neutrophil recruitment, a FPR2-dependent process that can be blocked by AnxA1-derived peptides [37]. Along these lines, in ovalbumin-induced atopic dermatitis-like skin lesions, AnxA1 is responsible for the production of allergen-induced immunoglobubin E, cytokines as well as the recruitment of inflammatory cells, in particular mast cells, to the lesion site [38]. Other studies identified mast cell stabilizers to treat ocular allergy, such as cromoglycate and nedocromil, to promote cellular AnxA1 release, which limits mast cell degranulation and the extend of the allergen-mediated allergic reactions [39,40]. Interestingly, AnxA1 not only impacts on neutrophil homeostasis in the presence of glucocorticoids, but also seems to modulate steady-state neutrophil maturation [31]. In efforts to clarify the role of exogenous AnxA1 for the hematopoietic system, administration of AnxA1, most likely via FPR-induced Ca^2+^ and MAPK signalling, promotes myeloid and granulocytic differentiation [41].

Adding further complexity, antigen presentation and T cell activation are also modulated by the AnxA1/FPR2 axis, with consequences for the adaptive immune response [21,33,42,43]. Of note, transgenic mice overexpressing AnxA1 exclusively in T lymphocytes led to an unexpected increase in anxiety, possibly due to an anxiogenic factor released by AnxA1 overexpressing T cells, which may contribute to increased susceptibility for mental disorders in patients with autoimmune diseases [44].

#### 2.1.1. AnxA1 and Progression of Chronic Diseases

In order to return local sites of inflammation to normal homeostasis, AnxA1 is now considered a pro-resolving mediator that can counteract pro-inflammatory response. In fact, administration of recombinant AnxA1 can delay or even regress the progression of chronic diseases, such as cardiovascular disease [22,45,46,47] (Table 1b). For instance, in several murine stroke models, AnxA1 administration mediated protective effects via FPR2 after cerebral ischemia/reperfusion (I/R) injury [48] or more recently, after spontaneous intracerebral hemorrhage [49], limiting further cerebral microvascular dysfunction and tissue damage. In addition, binding of AnxA1-derived peptides to FPR2 on neutrophils was demonstrated to regulate neutrophil-platelet aggregation, which contributed to attenuate cerebral inflammation [50].

AnxA1 administration to low density lipoprotein receptor (LDLR)—deficient mice that were fed a western-type diet attenuated progression of atherosclerotic plaques and was accompanied by reduced FPR2-dependent neutrophil rolling and adhesion to endothelial cells [51]. Conversely, enhanced atherosclerotic lesion formation and arterial myeloid cell adhesion was observed in apolipoprotein E (*apoE*)^−/−^ mice that lacked either AnxA1 or FPR2. Administration of the AnxA1-derived peptide Ac2-26 ameliorated this phenotype in the AnxA1 KO-mice but not in the FPR2 KO-mice, as judged by reduced atherosclerotic lesion size, lessened macrophage accumulation in lesions and a decreased FPR2-dependent recruitment of myeloid cells [32]. The protective capacity of AnxA1 was also documented in a model of restenosis, examining mechanical wire injury in high-fat diet (HFD)—fed AnxA1-deficient *apoE^−/−^* mice. In these animals, aggravated neointima development due to the accumulation and proliferation of macrophages was observed, suggesting that AnxA1 negatively regulates macrophage proliferation and its administration could serve to prevent restenosis after vascular damage [52].

Relevant for cardiac repair after myocardial infarction (MI), AnxA1 deficiency increased cardiac necrosis, inflammation, hypertrophy and fibrosis following MI and was accompanied by an impaired macrophage phenotype [53]. The reduced cardiac functionality in *Anxa1^−/−^* mice after MI could be restored by AnxA1 administration, overcoming compromised release of proangiogenic mediator vascular endothelial growth factor (VEGF) from cardiac macrophages, and markedly improving neovascularization and cardiac repair [53]. AnxA1-based therapies improved cardiac outcomes after MI also in other studies [54] (Table 1b).

#### 2.1.2. AnxA1-Derived Peptides and Anti-Inflammatory Response

As mentioned above, administration of full length AnxA1 greatly advanced efforts to explore the therapeutic potential of targeting FRP2 for anti-inflammatory strategies. Peptides of the N-terminal AnxA1 region, the most widely used being the Ac2-26 peptide, also mimic anti-inflammatory actions of full length AnxA1 [21,22,23,33,55,56]. The review articles listed here summarize a substantial amount of work demonstrating that exogenous administration of AnxA1 or Ac2-26 effectively limited or resolved inflammation in mouse models of stroke, myocardial ischemia, non-alcoholic steatohepatitis, rheumatoid arthritis, multiple sclerosis, colitis and asthma. AnxA1 deficiency was also detrimental in epithelial wound repair [11,57], lung fibrosis [58], obesity and insulin resistance [59], skin grafting [60], bacterial infection [61,62], as well as adrenal steroidogenesis in sepsis [63], or allergic conjunctivitis [64], and seems to have regulatory functions in early pregnancy [65] (Table 1a,b).

Recently, resolving AnxA1 functions were documented in a murine model of cardiotoxin-induced tibialis anterior injury. In this model, which is characterized by necrotic tissue damage and extensive macrophage activity, extracellular AnxA1 was delivered to the injured tissue through neutrophil recruitment and overexpressing macrophages, triggering FPR2-mediated macrophage skewing towards a pro-reparative phenotype, dampening of inflammation and ultimately, regeneration of skeletal muscle fibers [66]. It has yet to be determined how this might relate to notexin-induced injury, which implicated AnxA1 deficiency to interfere with cell–cell fusion during myofiber regeneration [67]. A protective function for AnxA1 was also reported for mechanical injury-induced corneal scarring [68], acute colitis [69], and gout inflammation [70] (Table 1c).

Ac2-26 also showed anti-inflammatory and neuroprotective effects in diseases related to the central nervous system, such as pilocarpine-induced status epilepticus [71], kainic acid-induced temporal lobe epilepsy [72], or cerebral inflammation in sepsis [73]. However, Ac2-26 administration in a mouse model of Alzheimer disease did not exert any beneficial effects [74], possibly due to limitations in its ability to cross the blood–brain barrier (Table 1d).

### 2.2. AnxA1 and Diabetes

In the context of diabetic complications, initial studies reported that female *Anxa1^−/−^* mice on the *Balb/c* background were more susceptible to weight gain and diet-induced insulin resistance, without showing significant changes in inflammation [59] (Table 1e). More recently, type 2 diabetic patients were found to exhibit increased AnxA1 plasma levels, which correlated with fatty liver index and elevated plasma cholesterol [34]. In these studies, a HFD in AnxA1^−/−^ mice on the *C57BL/6* background led to a more diabetic phenotype. Treatment of HFD-fed wildtype mice with recombinant AnxA1 attenuated the development of insulin resistance [34]. AnxA1 plasma levels in human type 1 diabetics were similarly found to be elevated [75]. This coincided with a worse diabetic phenotype and severe cardiac and renal dysfunction of streptozotocin (STZ)-induced type 1 diabetes in AnxA1^−/−^ mice. Strikingly, AnxA1 administration attenuated cardiac and renal complications in diabetic *AnxA1^−/−^* animals, and was associated with reduced activity of FPR2 signaling pathways [75]. In other studies, AnxA1 deficiency worsened pathological remodelling of the mesenteric vasculature in insulin-resistant, but not insulin-deficient animals [76], suggesting that AnxA1-based therapies could provide benefits for reducing vascular injury in diabetes. In support of this, a novel AnxA1-mimetic (compound 17b) provided vasoprotective effects in STZ-induced diabetic mice through the upregulation of vasodilating prostanoids [77].

Several other functions of AnxA1 may contribute to improve diabetic complications (Table 1e). For instance, exogenous AnxA1 enhanced glucose-stimulated insulin secretion of islet cells, which could be beneficial in islet transplantation strategies as a therapy for type 1 diabetes [47,78]. Furthermore, Ac2-26 application in diabetic wounds reduced neutrophil accumulation and facilitated M2 macrophage development, both anti-inflammatory and pro-repair effects required for wound closure [79]. On the other hand, it should be noted that when analyzing skin wound healing in AnxA1-deficient mice, wound inflammation, closure and formation of granulation tissue were not altered, and AnxA1 was proposed to possibly be dispensible for wound hemostasis and repair [80].

### 2.3. Nanotechnology-Based Approaches to Deliver AnxA1-Derived Peptides

As outlined above, the Ac2-26 peptide is the most widely used AnxA1-derived peptide to resolve inflammation. However, this peptide not only binds FPR2, but also FPR1 [23], and a novel peptide covering a larger region of the AnxA1 N-terminus (2-48) specifically bound FPR2 and reduced neutrophil recruitment during dermal inflammation [81]. Additionally, as oral delivery of peptides for therapeutic use are known to suffer from low bioavailability and metabolic liability, efforts to develop a more efficient and/or targeted delivery of AnxA1-derived peptides have been made (Table 1f). For example, Ac2-26 was packaged onto nanoparticles that target collagen type IV, which is highly enriched in atherosclerotic plaque. Advanced atheroslerotic lesions from mice receiving these Ac2-26 peptide-containing nanoparticles showed reduced lesion instability in a FPR2-dependent manner [82], indicating an efficient and localized delivery of the Ac2-26 peptide.

Similarly, delivery of oxidation-responsive and Ac2-26 containing nanoparticles to the inflamed colons of mice enabled site-specific release of the pro-resolving AnxA1-mimetic peptide for the treatment of inflammatory bowel disease [83]. Oral delivery of these oxidation-labile Ac2-26 containing nanoparticles effectively reduced inflammation in the gastrointestinal tract, and improved intestinal wound healing [83]. The fact that neutrophil-derived microvesicles contain AnxA1, which bind to FPR2 and contribute to resolve cartilage protection in arthritic mice [84], also support nano-based therapeutic strategies as a promising tool to deliver AnxA1 or AnxA1-mimetic peptides.

Other approaches include the fusion of AnxA1-derived peptides with a cell-penetrating peptide to protect against neuronal apoptosis after cerebral ischemia [85], or the grafting of a small bioactive AnxA1 peptide into a stable cyclic peptide scaffold for the reduction of inflammation in a mouse model for acute colitis [86]. Alternatively, the development of a doxorubicin-DNA aptamer conjugate that specifically recognizes AnxA1 on the surface of cancer cells significantly enhanced targeted therapy against tumours in vivo [87]. Likewise, coupling anticancer drugs to a peptide that recognizes AnxA1 on the tumour vasculature surface enabled drug delivery across the blood–brain barrier to suppress growth of brain tumours [88]. Finally, one report described the utilization of a monoclonal AnxA1 antibody to suppress the autoimmune syndrome associated with an abnormal AnxA1 expression on activated B and T cells in a mouse model of systemic lupus erythematosus (SLE), thus underscoring the need for a balanced expression of this immune modulator [89].

### 2.4. AnxA1 and Cancer

Over the last few years, a lot of studies have explored a variety of therapeutic roles for AnxA1 in cancer (Table 1g). For instance, chemotherapeutic agents such as anthracyclines and cyclophosphamide can trigger an anticancer immune response, with long-term beneficial effects beyond the initial chemotherapy. The underlying mechanism appears to be based on the release of AnxA1 from cancer cells upon chemotherapy. AnxA1 then binds to FPR1 on dendritic cells, which phagocytose and kill malignant cells, but also present tumour-associated antigens, which are then recognized and eliminated by cytotoxic T lymphocytes [90,91]. In support of this model, anticancer immunity after chemotherapy with anthracyclines was compromised in mice with tumours lacking AnxA1, or when the dendritic host cells lacked FPR1. This regulatory circuit may be tumour-type specific and relevant in breast, colorectal, lung, and kidney, but not gastric cancers. Other limitations to boost anticancer immunity in cancer patients via AnxA1 administration include the high prevalence of a loss-of-function FPR1 allele common in all ethnic groups, which is associated with poor prognosis, at least in breast cancer patients, after anthracycline-based chemotherapy [90,92].

In contrast, scenarios driven by the binding of AnxA1 to FPRs have associated high AnxA1 levels with poor patient survival and progression in metastatic and triple-negative breast cancers (TNBC) [93]. Along these lines, AnxA1-deficient mice displayed reduced breast cancer growth and enhanced survival, and it was proposed that AnxA1 binding to FPR2 on macrophages, influencing their polarization and infiltration of solid tumours, would be the underlying cause for enhanced tumour progression [94]. Alternatively, increased binding of AnxA1 released from cancer cells to FPR2 on regulatory T cells may impede anti-tumour immune responses [95]. Adding further complexity, secretion of AnxA1 from TNBC cells might induce autocrine signalling via FPR1 to further increase breast cancer cell aggressiveness and survival and could be blocked with cyclosporin A, a FPR1 inhibitor [96].

The de-regulated signaling pathways in cancers triggered by extra- or intracellular AnxA1 activities have yet to be fully elucidated [90,91,92,93,94,95,96]. Phosphoproteomics from AnxA1-deficient mammary gland epithelial cells identified up- and downregulation of several signaling pathways that modulate cell motility and could contribute to breast cancer initiation [97]. Mechanistically, AnxA1 was shown to control epidermal growth factor receptor (EGFR) receptor signaling and trafficking [98], which is probably modified through AnxA1 phosphorylation [99]. In pancreatic cancer, AnxA1 depletion conferred a less aggressive phenotype and reduced liver metastasis independent of FPR pathways [100]. In a mouse model for multiple myeloma, AnxA1 depletion improved bortezomib treatment [101], but in nasopharyngeal carcinoma xenografts, loss of AnxA1 enhanced radiotherapy resistance [102] (Table 1g).

### 2.5. Intracellular AnxA1 Activities

Besides the AnxA1/FPR axis, the AnxA1 KO-strain also revealed intracellular AnxA1 activities to modulate inflammation and other disease-related activities (Table 1h). For instance, *Anxa1^−/−^* macrophages displayed an altered activity of signaling pathways and the NOD-, LRR- and pyrin domain-containing protein 3 (NLRP3) inflammasome, both affecting inflammatory cytokine production, such as interferon-β and tumour necrosis factor α (TNFα), but also release of lipid mediators [103,104,105,106]. Defects in the secretory pathway of mesenchymal stromal cells from the AnxA1 KO-mice might explain the inability of the secretome from these cells to stimulate glucose-induced insulin secretion of pancreatic β cells [47,78].

In addition, nuclear translocation of AnxA1 [107], as well as AnxA1 affecting transcriptional activation, mRNA transport and stability has been described [103,104]. Intracellular AnxA1 activities may also explain the upregulation of cyclooxygenase (COX) and cytoplasmic phospholipase A2 (cPLA_2_) observed in *Anxa1^−/−^* fibroblasts, probably driving enhanced and glucocorticoid-insensitive eicosanoid production in the *Anxa1^−/−^* animals [24,108].

*Anxa1^−/−^* fibroblasts also revealed the critical role of AnxA1 in the biogenesis of a subpopulation of internal vesicles in multivesicular bodies (MVBs) [109]. In these studies, AnxA1 was transported on to intraluminal MVB vesicles containing EGFR and was tyrosine-phosphorylated upon EGFR activation. EGF-stimulated inward vesiculation was abolished in *Anxa1^−/−^* fibroblasts, whereas basal inward vesiculation remained intact. Hence, physical association and tyrosine phosphorylation of AnxA1 for EGF-stimulated and EGFR-containing intraluminal vesicle formation within MVBs appears critical [109]. In addition, AnxA1 contributes to the establishment of membrane contact sites, which is likely relevant for trafficking of receptors and transfer of ions and lipids such as cholesterol, between endosomal and other compartments [110]. Additionally, *Anxa1^−/−^* mice are partially protected against influenza A virus infection, involving reduced uptake and exit of internalized virus from late endosomes/MVBs [111]. Yet, this protective effect of AnxA1 against viral infection also requires the AnxA1/FPR2 signaling axis to increase alveolar macrophages, which are decisive factors in the host defence against pathogens [112] (Table 1h).

## 3. AnxA2

In mice, AnxA2 is highly expressed in the lung, pancreas, colon, ileum and adrenal tissues, while low AnxA2 levels are found in spleen, testis, kidney and liver [114]. In endo- and epithelial cells, monocytes, macrophages, and many other cell types, AnxA2 regulates microdomain formation and membrane repair at the plasma membrane, trafficking along endo- and exocytic pathways, as well as RNA export from the nucleus [115,116]. Multiple membrane-related AnxA2 activities involve interactions with the actin cytoskeleton and phosphatidylinositol-4,5-bisphosphate, contributing to cell growth, differentiation, apoptosis and migration [1,5,6,116].

Notably, AnxA2 forms a heterotetramer with p11 (S100A10), a member of the S100 protein family, and upon Src kinase-mediated phosphorylation of AnxA2 at tyrosine 23, the intracellular AnxA2/p11 complex is translocated to the outer membrane leaflet. At the cell surface, the heterotetrameric AnxA2/p11 complex acts as a scaffold that modulates cell surface presentation of receptors and ion channels [117,118]. Indeed, the critical role of the AnxA2/p11 complex for the cell surface presentation of serotonine receptors has been validated in AnxA1-, p11-KO and other mouse models of depression [115,119,120].

### 3.1. AnxA2 and Vascular Homeostasis and Angiogenesis

On endothelial cells, extracellular AnxA2/p11 serves as a docking site for plasminogen and tissue plasminogen activator (tPA), which promote plasmin generation and vascular fibrinolysis. The AnxA2/p11-mediated plasminogen activation has been extensively discussed and we recommend several reviews for further reading [115,116,119,120,121].

AnxA2-deficient mice [122] (Table 2a) provided the first in vivo evidence that AnxA2 mediates fibrin clearance, as the AnxA2^−/−^ mice showed substantial deposition of fibrin and after induction of acute carotic artery thrombosis, displayed increased thrombosis. Mechanistically, Src kinase and protein kinase C (PKC) were required for AnxA2/p11 translocation to the cell surface in the carotid artery injury model [123]. Further supporting a connected role for AnxA2 and p11 in fibrinolysis, de-regulated fibrinolysis was also observed in the p11 KO-mice [124]. This coincides with reduced p11 levels in the *Anxa2^−/−^* mice, most likely due to increased p11 ubiquitination and degradation in these animals [125].

In other studies, *Anxa2^−/−^* mice revealed AnxA2-mediated functions in vascular homeostasis and angiogenesis, such as the formation of new blood vessels [115,122,126]. In a mouse model for diet-induced hyperhomocysteinemia, which is implicated in thrombotic and atherosclerotic disease, fibrin accumulation and defects in neoangiogenesis were similar to those observed in the *Anxa2^−/−^* mice [127]. Interestingly, AnxA2 isolated from mice fed a high methionine diet failed to bind tPA and activate plasminogen and could only be restored upon administration of exogenous AnxA2 [127]. In ischemic cerebral disease, recombinant AnxA2 amplified tPA-mediated thrombolysis to prevent stroke [128]. Yet, although inhibition of plasmin generation has therapeutic potential in atherosclerosis, AnxA2 deficiency in *apoE^−/−^* mice did not reduce lesion development [115]. The latter observation is challenged by AnxA2 deficiency suppressing atherogenic integrin α5 signalling caused by oscillary shear stress, which reduced the development of atherosclerosis in apoE^−/−^ mice at regions with disturbed blood flow [129]. Recently, recombinant AnxA2 ameliorated fibrinolytic dysfunction in a mouse model lacking the exchange protein directly activated by cAMP (EPAC), which is required for fibrinolysis on the surface of vascular endothelial cells [130] (Table 2a).

#### 3.1.1. AnxA2 and Pulmonary Vasculature

In the pulmonary vasculature, endothelial cells from *Anxa2^−/−^* mice display reduced barrier function and develop pulmonary edema and neutrophil infiltration in the lung parenchyma in response to alveolar hypoxia. Pulmonary vascular integrity does not seem to require the AnxA1/p11 tetramer, but AnxA2 complex formation with vascular endothelial cadherin and its tyrosine phosphatases, to prevent vascular leak during alveolar hypoxia [131] (Table 2b). In addition, AnxA2 can contribute to lung injury and fibrosis by augmenting factor Xa fibrogenic activity [132]. Interestingly, within the pulmonary vasculature, AnxA2 has now been found responsible for the severe side effects caused by bleomycin, a clinically potent anticancer drug for a large variety of cancers. Bleomycin binds AnxA2, which impedes transcription factor EB mediated autophagy, leading to the induction of pulmonary fibrosis. Underlying mechanism involve bleomycin-induced NF-kappaB (NFκB) inflammatory pathways leading to AnxA2 secretion. Strikingly, these toxic side effects can be overcome by an AnxA2 peptide inhibitor (TM601) or AnxA2 deficiency in mice, alleviating bleomycin-induced pulmonary inflammation and fibrosis [132,133,134].

In addition, a critical in vivo function of AnxA2 in the secretory pathway affects pulmonary function (Table 2b). *Anxa2^−/−^* displayed reduced exercise tolerance and impaired lung tissue elasticity that resembles the phenotype of collagen VI (COL6) deficiency, which anchors basement membranes to other collagen fibers [135]. Indeed, the *Anxa2^−/−^* lung basement membrane lacked COL6, which accumulated and mislocalized in the Golgi apparatus [135]. This phenotype of the *Anxa2^−/−^* mice correlated with the association of AnxA2 with COL6, but also two SNARE (soluble NSF attachment protein receptor) proteins, synaptosomal-associated protein 23 (SNAP23) and vesicle-associated membrane protein 2 (VAMP2), in secretory vesicles from bronchial epithelial cells (Table 2b). This is in line with previous reports identifying AnxA2 to regulate exocytosis via several SNAREs, including SNAP23 and VAMP2 [136,137]. This secretory function of AnxA2 may extend to other disorders associated with COL6 deficiency [135] or exocytic events driving cancer progression [138,139,140].

#### 3.1.2. Other AnxA2-Related Aspects in Vascular Homeostasis

Other AnxA2-related aspects in vascular homeostasis and angiogenesis include the high AnxA2 antibody titers associated with thrombotic complications in cerebral venous thrombosis [115,119,120] as well as the antiphospholipid syndrome [141,142]. AnxA2 upregulation also correlated with hyperfibrinolysis in acute promyelocytic leukemia [119,120]. Administration of recombinant AnxA2 increased neovascularization and inflammation in mice with collagen-induced arthritis via binding to an AnxA2 receptor described previously [143] and subsequent activation of the Hedgehog pathway [144]. On the other hand, lentiviral AnxA2 receptor overexpression suppressed neovascularization in the mouse aortic ring and matrigel plug assays [145]. The role of AnxA2 in vascular homeostasis extends to endothelial permeability and integrity at the blood–brain barrier after cerebrovascular injury, with AnxA2-deficient mice displaying increased inflammation and reduced expression of cerebral endothelial junctional proteins [146,147]. For further reading of AnxA2-related aspects in vascular homeostasis and angiogenesis we recommend several excellent reviews [115,116,119,120,121,142] (Table 2c).

### 3.2. AnxA2 and Lipid-Related Disorders

Several studies investigated AnxA2 in mouse models for lipid-related disorders (Table 2d). Possibly relevant for hypercholesterolemic settings, AnxA2 or the AnxA2/p11 complex inhibited proprotein convertase subtilisin/kexin-9 (PCSK9)-mediated LDLR downregulation, leading to elevated LDLR levels at the cell surface [114,148]. *Anxa2^−/−^* mice displayed elevated LDL-cholesterol and circulating PCSK9 levels, while LDLR levels were reduced in extrahepatic tissues. Vice versa, adenoviral AnxA2 overexpression elevated hepatic LDLR levels in vivo [114]. The association of LDL-cholesterol levels with *Anxa2* gene variants [149] support modulation of AnxA2 levels as a potential cholesterol-lowering strategy. Applicable for obesity and the metabolic syndrome, AnxA2 depletion attenuated obesity-induced insulin resistance through suppression of NFκB signaling [150]. Furthermore, in vascular endothelial cells of white adipose tissue (WAT), AnxA2 forms a complex with prohibitin and the fatty acid transporter CD36. *Anxa2^−/−^* mice exhibited defective fatty acid uptake by the WAT endothelium and adipocytes, making this regulatory circuit a new target for metabolic intervention in obesity [151] (Table 2d).

### 3.3. AnxA2 and Cancer

A substantial number of studies in recent years examined AnxA2 in cancer growth and progression in vivo, identifying diverse AnxA2 functions in tumourigenic settings (Table 2e).

In mouse models for pancreatic ductal adenocarcinoma (PDAC), AnxA2 KO-animals displayed a substantial reduction in PDAC tumour invasion and metastasis. Src-dependent AnxA2 tyrosine 23 phosphorylation and cell surface translocation, as well as AnxA2 regulating the secretion and autocrine signaling of semaphorin 3D (SEMA3D) contribute to PDAC invasion and metastasis [138]. The reduced SEMA3D secretion upon loss of AnxA2 also lowered the incidence of perineural invasion and metastasis of orthotopic pancreatic tumours in mice [152]. PDAC metastatic behaviour is strongly influenced by stromal cells in the tumour microenvironment and pharmacological inhibition of signaling events initiated by factors released from stroma cells, including hepatocyte growth factor, insulin-like growth factor 1 or tenascin C, reduced AnxA2 phosphorylation and metastatic potential of PDAC tumours [139,140,153]. In fact, stromal AnxA2 expression has potential as a predictive biomarker for survival outcomes in PDAC [154]. The therapeutic potential of targeting AnxA2 in PDAC was further emphasized with the recent development of an AnxA2-targeting vaccine approach, which in combination with immune checkpoint inhibitors, led to survival benefits in PDAC mouse models [155]. Additional evidence for roles of AnxA2 in the tumour microenvironment came from the inoculation of prostate cancer cells with bone marrow stromal cells from the AnxA2 KO-mice. This approach revealed that AnxA2 binding to stromal-derived factor 1 strongly influenced recruitment, growth and survival of prostate cancer cells in the bone marrow microenvironment [156] (Table 2e).

#### Therapeutic Potential of AnxA2 in Cancer

Several animal studies proposed multiple roles for AnxA2 in brain cancers. In pediatric neuroblastoma, AnxA2 depletion attenuated NFκB activity and enhanced anticancer drug sensitivity, which improved chemotherapy in a xenograft model for neuroblastoma [157]. Likewise, AnxA2-mediated NFκB signaling contributed to tumourigenicity in xenograft models for glioblastoma multiforma (GBM) [158], with AnxA2 depletion strongly inhibiting GBM tumour growth and improving survival [159]. In the latter studies, AnxA2-mediated phosphorylation of signal transducer and activator of transcription 3 (STAT3) increased expression of microRNA miR155 to promote epithelial-to-mesenchymal transition (EMT) in glioma [159]. Other downstream targets of the AnxA2-STAT3 axis to promote growth and phenotypic transition in GBM include the oncostatin M receptor [160] and the cyclin D1 pathway [161] (Table 2e).

In breast cancer, intra- and extracellular AnxA2 activities and locations, including exosomes [162], have become promising therapeutic targets. AnxA2 antibody-conjugated and curcumin-loaded nanoparticles effectively accumulated in tumours, providing sustained release of curcumin with potential to reduce metastatic breast cancer progression [163]. A monoclonal AnxA2 antibody not only allowed monitoring EMT in breast and ovarian cancers, but also provided antibody-dependent cell toxicity and efficient killing when conjugated to cytotoxic drugs and expressed as a chimeric immunoglobulin G1 [164,165,166]. Other beneficial effects of AnxA2 antibodies include the inhibition of neoangiogenesis in TNBC xenograft models, which involves reduced plasmin generation as well as loss of AnxA2 tyrosine 23 phosphorylation [167,168]. The latter requires interaction with Src and Rack1 kinases, and could be responsible for the acquirement of drug resistance in aggressive breast cancers [169]. In support of this, AnxA2 depletion improved the suppressive xenograft growth effect of Src kinase and VEGF inhibitors in esophageal cancers [170].

In fact, AnxA2 contributes to the development of resistance to cisplatin and tyrosine kinase inhibitors targeting EGFR in non-small cell lung cancers [171,172]. Vice versa, disruption of AnxA2/p11 interaction with small molecule inhibitors or AnxA2 antibodies reduced acute lymphoblastic leukemia proliferation and sensitized tumour cells to chemotherapy [173].

Although data from p11 KO-mice often supports findings that AnxA2/p11-dependent plasmin generation is involved in tumour progression [115,116,121], it should be noted that p11 has plenty of other tumourigenic functions that appear rather unrelated to AnxA2 [174,175]. It would also go beyond the scope of this review to discuss in detail cancer-related animal studies that identified upstream regulators of AnxA2 expression or activity [176,177,178]. However, other studies, for example in glioma, suggest that not only tetrameric, but also monomeric AnxA2 might serve as a strategy to develop it as a vaccine adjuvant to increase anti-tumour immunity [179] (Table 2e). Alternatively, a novel RNA nanoparticle harboring an AnxA2 aptamer was developed to target ovarian cancer for doxorubicin delivery [180]. Similary, a DNA aptamer that binds AnxA2 on cancer cells could become a tool to diagnose and treat multiple myeloma [181]. Along these lines, a cyclic octapeptide labeled with a near-infrared dye selectively binding phosphorylated AnxA2, which is highly expressed in a wide range of solid tumours, provides opportunity for tumour imaging and localized drug delivery [182]. Alternatively, treatment of tumour cells with a cytosol-targeting small peptide that binds to intracellular AnxA2 showed a reduced capacity to colonize lungs in several mouse models [183] (Table 2e).

### 3.4. AnxA2 and Infection

Several recent studies addressed roles for AnxA2 in response to bacterial and viral infections, some of those identifying roles for AnxA2 in membrane organization and endocytic transport (Table 2f). For instance, pathogen recognition and activation of innate immunity in macrophages requires Toll-like receptor 4 (TLR4) activation at the plasma membrane and on endosomes. Remarkably, in *Anxa2^−/−^* mice enhanced TLR4 signaling and reduced TLR4 endocytosis was associated with an increased susceptibility to bacteria-induced pulmonary inflammation [184].

During fungal infection (*Cryptococcus*) of macrophages, AnxA2 controls phagocytosis and non-lytic exocytosis of the pathogen. Compromised fungal uptake, propagation and release, together with an enhanced inflammatory response, probably explains that *Anxa2^−/−^* mice died more rapidly than control animals after fungal infection [185].

In studies addressing autophagy, vesicles isolated from dendritic cells of *Anxa2^−/−^* mice contained reduced amounts of phosphatidylserine (PS) and phosphatidylinositides. These vesicles generate phagosomes for encapsulation of organelles and biomolecules, followed by their degradation in endo-/lysosomes. Phagosome biogenesis and maturation in dendritic cells from the *Anxa2^−/−^* mice was strongly reduced [186]. Autophagy can also serve as a defence mechanisms against bacterial infection, and was de-regulated in a mouse infection model using *Anxa2^−/−^* animals [187].

In a mouse model for Alzheimer’s disease, AnxA2 interacts with phosphorylated presenilin 1 and the SNARE protein VAMP8, the latter binding to the autophagosomal SNARE syntaxin 17. This ultimately facilitates fusion of autophagosomes with lysosomes, thereby increasing b-amyloid degradation, which has therapeutic potential for Alzheimer’s disease [188].

Tick saliva contains effector molecules that inhibit host immunity and facilitate pathogen transmission. One of the tick proteins, sialostatin L2, binds AnxA2, which then impairs the formation of the NLRC4 inflammasome, responsible for the maturation and secretion of pro-inflammatory cytokines. Accordingly, AnxA2-deficient mice were more succeptible to *Anaplasma phagocytophilum* infection, leading to splenomegaly, thrombo- and monocytopenia [189]. On the other hand, during the pathophysiology of sepsis, *Anxa2^−/−^* mice showed increased production of pro-inflammatory IL-17 and reactive oxygen species, increased neutrophil infiltration, but reduced bacterial clearance and animal survival [190]. While these studies rather implicate intracellular AnxA2 functions, during the adhesion and invasion of uropathogenic *Escherichia coli* to bladder epithelial cells, AnxA2 was identified as the receptor of the bacterial protein YadC, which participates in binding to bladder epithelial cells [191]. Inhibition of AnxA2 using the Ca^2+^-chelator BAPTA-AM or compound A-05, which inhibits the AnxA2/p11 complex [192], attenuated *E.coli* infection in vivo [191]. Likewise, AnxA2 on the surface of vascular endothelial cells served as a docking site for *Rickettsia*, with AnxA2 deficiency blocking *Rickettsia* adherence to the luminal surface of blood vessels in vivo [193] (Table 2f).

### 3.5. AnxA2 and Muscle Repair

Two recent studies linked AnxA2 with myofiber repair and inflammation in muscle [194,195] (Table 2g). Muscle repair after sarcolemmal damage requires dysferlin, and similar to dysferlin deficiency, lack of AnxA2 in myofibers was associated with poor sarcolemma repair and a progressive age-dependent decline in muscle function. Yet, AnxA2 deficiency did not cause chronic inflammation that is commonly observed upon loss of dysferlin. Moreover, AnxA2-depleted mice showed extensive myofiber regeneration after laser injury, but not degeneration or adipogenic replacement, making AnxA2 a novel target to treat dysferlinopathy [194]. Indeed, lack of AnxA2 prevented adipogenic replacement of myofibers, while exogenous AnxA2 enhanced muscle loss in mouse models for limb girdle muscular dystrophy 2B, which is caused by dysferlin mutations [195].

### 3.6. Other AnxA2-Related Biological Activities

Although the underlying mechanism remain to be fully clarified, roles for AnxA2 in actin polymerization and secretion might be involved in preeclampsia, a pregnancy-specific disorder, as AnxA2 deficiency impaired decidualization of endometrial stromal cells and the uterine microenvironment that promotes embryo implantation and placentation [196]. Alternatively, AnxA2 might act as an adhesion molecule at the endometrial epithelium during embryo implantation [197] (Table 2h).

Finally, in vivo evidence that links AnxA2 with ion channel regulation [1,2] includes AnxA2 being a ligand for the transient receptor potential ion channel A1 (TRPA1), which is responsible for the detection of harmful chemicals, tissue damage and chronic pain. *Anxa2^−/−^* mice displayed increased surface levels of TRPA1 on sensory neurons, which correlated with enhanced TRPA1-dependent pain in these animals [198].

## 4. AnxA3

AnxA3 is found in many cell types and preferentially expressed in the spleen, lung and reproductive organs with roles in membrane transport, ion transport, cytoskeletal interactions, cell signaling, inflammatory responses, endothelial cell migration, adipocyte differentiation, and vascular development [199,200,201,202,203,204].

### 4.1. AnxA3 and Cancer

Several studies examined AnxA3 loss- or gain-of-function in xenograft models, implicating AnxA3 depletion as a tool to improve anticancer drug sensitivity (Table 3). For instance, downregulated AnxA3 levels improved cisplatin sensitivity in ovarian cancer [205]. Similarly, AnxA3 silencing in MDA-MB-231 breast cancer cells strongly reduced lung metastasis with increased sensitivity towards doxorubicin, possibly through NFκB inhibition [200]. Others reported AnxA3 depletion in MDA-MB-231 cells to strongly reduce xenograft growth and angiogenesis in the tumour microenvironment [206]. AnxA3 silencing using miR382, which was accompanied by phosphoinositide-3-kinase/(PI3K)/protein kinase B (Akt) downregulation, reduced EMT and lymph node metastasis of pancreatic cancer cells [207]. In A549 lung adenocarcinoma cells, AnxA3 deficiency reduced MAPK signaling and decreased expression of metalloproteases and E-/N-cadherin, thereby reducing xenograft growth, as well as metastasis to lung, liver and brain after tail vein injection [208]. In lung cancer, cancer associated fibroblasts (CAFs) increased AnxA3 levels in the neighbouring cancer cells, which correlated with increased cisplatin resistance. In contrast, AnxA3 knockdown in this model increased cisplatin sensitivity, an observation that may add to overcome drug resistance in lung cancer patients [209]. In other studies, elevated AnxA3 levels suppressed PKCδ/p38 MAPK-dependent onset of apoptosis and autophagy [210] and might be a target for immunotherapy in hepatocellular carcinoma [201]. Along these lines, AnxA3 monoclonal antibodies sensitized hepatocellular carcinoma to the tyrosine kinase inhibitor sorafenib [210]. Finally, AnxA3 depletion in microglia alleviated bone cancer-induced pain through inhibition of hypoxia-inducible factor-1a/VEGF-dependent signaling [211] (Table 3).

## 5. AnxA4

AnxA4 is expressed prominently in secretory epithelia of the lung, intestine, stomach and kidney [212]. Out of the three AnxA4 mRNAs (AnxA4a-c), AnxA4a is broadly distributed, while AnxA4b and AnxA4c are only found in the digestive track and solitary chemosensory cells, respectively [213]. After Ca^2+^ elevation, AnxA4 translocates to the plasma membrane or the nuclear membrane, but also has the ability to oligomerize and participate in Ca^2+^-dependent aggregation of vesicles or modulate the mobility of membrane-associated proteins [214,215,216]. While the latter may implicate a role in repair mechanisms, administration of pharmacological AnxA4 concentrations caused bleeding in skin wound repair [80]. AnxA4a-deficient urothelium did not reveal alterations in bladder function [217], but airway epithelial cell migration was impaired upon AnxA4 depletion, with consequences for airway branching morphogenesis during lung development [218].

In cardiomyocytes from adult AnxA4 KO-mice, β-adrenoreceptor (β-AR) agonists more potently increased cAMP levels, which coincided with enhanced cardiac contraction force in the heart [219]. The underlying cause involves the inhibitory action of AnxA4 on adenylyl cyclase 5, which controls conversion of ATP into cAMP, an activity that was recently mapped to the N-terminus of AnxA4 [220] (Table 4).

### 5.1. AnxA4 in Cancer

AnxA4 is upregulated in various epithelial cancers, and AnxA4 knockdown reduced xenograft growth of gallbladder cancer cells, possibly by downregulating oncogenic NFκB signaling pathways [221]. Likewise, AnxA4 depletion decelerated the in vivo growth of tumours derived from basal-like breast cancer cell lines. In this model, AnxA4 interacted with another annexin, AnxA1, to activate Janus kinase-STAT3 signaling [222].

Several studies examined AnxA4 in the context of anticancer drug resistance (Table 4). In xenografts from endometrial carcinoma cells, AnxA4 overexpression conferred cisplatin resistance, possibly by interacting with the copper transporter ATP7A, and promoting its ability to efflux platinum drugs [223]. Vice versa, AnxA4 depletion increased sensitivity to platinum-based drugs in vivo in a Ca^2+^-dependent manner [224]. Finally, chemoresistance of lung cancer cells correlated with AnxA4 translocation to the plasma membrane. Blocking AnxA4 translocation with a heptapeptide derived from the tumour suppressor FHIT restored the chemosensitivity to paclitaxel [225].

## 6. AnxA5

AnxA5 is the most abundant annexin in almost all cells and tissues, except neurons [1,226] (Table 5). In cells, AnxA5 is associated with the plasma membrane, nucleus, Golgi, endoplasmic reticulum, late endosomes/lysosomes, phagosomes, and mitochondria, often in a Ca^2+^-dependent manner and linked to the regulation of membrane transport, Ca^2+^ signaling, ion channels, Ca^2+^-influx, cell cycle, apoptosis and phagocytosis [227,228,229,230,231,232,233]. In addition, substantial amounts of AnxA5 are found extracellularly, and due to its strong binding affinity towards PS in the outer leaflet of the plasma membrane, AnxA5 is now a widely used diagnostic tool to detect apoptotic cells in various disease settings [226] (Table 5b–e and details below).

### 6.1. AnxA5 and Differentiation

Given the abundant AnxA5 levels in many tissues, several studies utilized the AnxA5 KO-mice to address possible roles in development. Indeed, the targeted disruption and replacement with an *Anxa5-lacZ* fusion gene enabled detailed AnxA5 expression analysis during embryonal mouse development, identifying *Anxa5-lacZ* expression specifically in populations of perivascular cells of non-skeletal mouse tissues, with critical roles in initial steps of angiogenesis differentiation and maturation of endothelial cells by pericytes [234,235]. However, and despite high AnxA5 levels in cartilaginous tissues and bone, the AnxA5 KO-mouse lacked any defects in the calcification process during skeletal development [236] (Table 5). This led to speculations that other annexins, in particular AnxA6, could compensate for AnxA5 deficiency [236,237]. Yet, double KO-mice deficient of AnxA5 and AnxA6 (*Anxa5^−/−^/Anxa6^−/−^)* or mice lacking both AnxA5 and AnxA6 as well as collagen X showed normal skeletal development [238,239], indicating these annexins not being essential for the calcification of the growth plate cartilage (Table 5a). Nevertheless, more recently, AnxA5 deficiency caused increased bone overgrowth at the enthesis, and it was concluded that AnxA5 prevented bone overgrowth triggered by mechanical forces and modulated expression of mineralization regulators [240].

Likewise, although being the most abundant membrane-associated protein in stereocilia, AnxA5 deficiency was dispensible for hair-bundle development and function [241] (Table 5a).

### 6.2. AnxA5 and Anticoagulation

AnxA5 is most abundant in the placenta and associated with thrombophilia-related complications in pregnancy [242]. Although AnxA5 deficiency was initially not considered to interfere with embryonal development, in later studies reduced litter size, increased foetal loss and placental thrombosis of maternally homozygous progeny were reported [243]. Yet, *Anxa5^−/−^* mice exhibited regular estrous cycle and ovulation numbers [243], although altered pituitary hormone expression patterns were observed [244,245]. Interestingly, administration of heparin to prevent thrombi formation in the placental circulation reduced pregnancy loss in the AnxA5 KO-mice (Table 5b). Alternatively, administration of Zinc^2+^ restored anticoagulation, upregulated AnxA5 levels in the placenta, and showed a trend to increase litter size in *Anxa5^−/−^* mice [246]. Moreover, fusion of AnxA5, with strong affinity for outer leaflet PS, with echistatin, which binds to the integrin α_IIb_β_3_ receptor on platelets, showed potential as antithrombotic reagent that specifically targets platelets in thrombogenesis sites [247]. Based on the property of AnxA5 to recognize PS on activated platelets, AnxA5-conjugated micelles have been developed to enable targeted delivery of drugs that promote thrombolysis [248] to alleviate the risk of hemorrhage that occurs after administration of thrombolytic agents.

In addition, the anticoagulant AnxA5 activity has implications for other diseases, as administration of wildtype AnxA5, but not an AnxA5 mutant version unable to bind PS, could overcome dysfunctional coagulation and inhibited thrombin formation in septic animals [249]. Mechanistically, the ability of AnxA5 to form two-dimensional arrays on PS-rich membranes in a Ca^2+^-dependent manner appears critical [242,250]. However, skin wound inflammation and closure were not altered in *Anxa5^−/−^* mice [80]. In fact, increased levels of AnxA5 in the alveolus may play a detrimental role in alveolar epithelial wound healing, promoting lung inflammation and fibrosis [251]. Nevertheless, AnxA5-based approaches could become a suitable tool to overcome poor obstetric outcomes caused by antiphospholipid syndrome, preeclampsia and SLE in humans [252] (Table 5b,c).

### 6.3. AnxA5 and Cardiovascular Disease

The ability of AnxA5 to bind cell surface PS on apoptotic cells has proven valuable for the diagnosis of atherosclerosis [226] (Table 5d). However, a comparison of radiolabeled (^99m^TC) AnxA5 with duramycin in HFD-fed *apoE^−/−^* KO-mice identified ^99m^TC-duramycin to be more suitable for the quantification of vulnerable atherosclerotic plaque [253]. These technical limitations might have contributed to the inability to detect any apoptotic changes in the aortic arch of *apoE^−/−^* mice treated with low dose radiation, which is considered to protect against atherosclerosis, when using ^99m^TC-labeled AnxA5 for autoradiographic analysis [254]. On the other hand, AnxA5-conjugated gold nanoparticles helped to better target localized apoptotic macrophages and diagnose vulnerable atherosclerotic plaque in the HFD-fed *apoE^−/−^* mouse model [255].

The therapeutic potential of recombinant AnxA5 binding to cell surface PS on apoptotic cells and thereby providing anticoagulant, but also anti-apoptotic and anti-inflammatory activities was shown in several studies (Table 5d). In *apoE^−/−^* mice fed a western style diet, subsequent AnxA5 administration reduced recruitment and activation of monocytes to the inflamed lesion site, thereby reducing plaque inflammation [256]. Moreover, when AnxA5 was administered simultaneously with the western style diet, reduced apoptotic rates contributed to the significantly reduced plaque size [257]. Using hypercholesterolemic ApoE*3 Leiden mice as a model for MI-reperfusion injury, AnxA5 treatment strongly reduced infarct size and improved cardiac function, most likely via the attenuation of the post-ischaemic inflammatory response [258]. Moreover, administration of AnxA5 fused to stromal-derived factor 1, a cytokine that protects the heart from ischaemic injury, accumulated at the myocardium, probably via binding to PS on apoptotic cells, and provided cardioprotection, as judged by attenuated apoptosis, enhanced angiogenesis, reduced infarcted size and improved cardiac function after MI [259]. Finally, AnxA5 administration improved steatosis, inflammation and fibrosis in nonalcoholic steatohepatitis [260]. Interestingly, the underlying mechanism in the latter studies might not involve AnxA5 binding to PS, but AnxA5 interacting with pyruvate kinase 2, leading to metabolic reprogramming from glycolysis to oxidative phosphorylation in macrophages [260] (Table 5d).

### 6.4. AnxA5 and Cancer

The ability of AnxA5 to recognize PS exposed by apoptotic cells has been very valuable in cancer-related studies (Table 5e). It would go beyond the scope of this review to list all studies that utilized AnxA5 as a read-out to monitor apoptosis, and only research that specifically examined the contribution of AnxA5 to oncogenesis is summarized here.

In hepatocarcinoma, AnxA5 overexpression promoted in vivo tumour malignancy and lymphatic metastasis [261], most likely through MAPK and Rac1 signaling pathways [262]. Likewise, AnxA5 upregulation promoted tumourgenicity of glioma cells in nude mice [263]. On the other hand, AnxA5 knockdown impeded tumourigenesis in subcutaneous xenografts from renal cell carcinoma [264], with the latter two studies suggesting an involvement of PI3K/Akt signaling and implicating AnxA5 as a potential biomarker in glioma and renal cell carcinoma. In non-small cell lung cancer, drug resistance to first-line EGFR tyrosine kinase inhibitors is common and AnxA5 depletion restored gefitinib sensitivity by promoting G2/M cell cycle arrest, providing opportunity to overcome this major clinical challenge [265].

In contradiction to the abovementioned tumour models, AnxA5 high-affinity binding to PS also inhibited engulfment of apoptotic cells by macrophages, which increased the immunogenicity of tumour cells undergoing apoptosis. Indeed, treatment of melanoma xenografts with AnxA5 reduced tumour growth, increased necrosis in tumour tissues and most strikingly, inhibited angiogenesis by downregulating VEGF production [266]. Hence, targeted delivery of AnxA5 could be utilized to selectively inhibit tumour growth and progression. Indeed, intra-tumoural release of AnxA5 from intravenously injected nanoparticles effectively blocked phagocytosis of apoptotic cells, which then triggered a strong cytotoxic T cell response and immunological memory that led to complete tumour regression in 50% of mice with orthotopic breast tumours [267]. Likewise, AnxA5 administration lessened the immunosuppressive properties of the tumour microenvironment generated by cisplatin treatment and enhanced the immunogenicity and anti-tumour efficacy in a preclinical tumour model with a defined tumour-specific antigen (TC-1 tumour model) [268,269]. Along these lines, fusion of AnxA5 with the peptide-major histocompatibility complex strongly augmented lymphocyte response, which could serve to improve immune modulation for cancer therapy [270]. In a mouse model for ovarian cancer, the delivery of AnxA5 fused to an enzyme that converts selenomethionine to toxic methylselenol, in combination with anti-CD73 and anti-Ox40 as immunostimulants, strongly decreased tumour burden in mice with orthotopic metastatic ovarian cancers [271] (Table 5f).

### 6.5. AnxA5 and Imaging

As molecular imaging techniques using radiolabeled AnxA5 provided opportunity to more rapidly assess apoptosis in disease or upon therapy, efforts over the last few years improved the imaging methodology based on AnxA5-derived tools to monitor apoptosis in vivo (Table 5d–f). As mentioned above, ^99m^TC radiolabeled AnxA5 and AnxA5-conjugated gold nanoparticles were valuable diagnostic imaging tools in mouse models for atherosclerosis [253,254,255] (Table 5d). AnxA5 dual-labeled with ^99m^TC and Alexa Fluor 568 allowed detection of ethanol-induced cell death in the femur muscle but failed to visualize cell death in a murine model for stroke [272]. Imaging of apoptosis using ^111^In- and DOTA-peptide -labeled AnxA5 monitored efficacy of suicide gene therapy after treatment with ganciclovir in NG4TL4 sarcoma expressing herpes simplex virus thymidine kinase [273]. Drug-induced apoptosis in tumour-bearing mice was also detectable using AnxA5-conjugated ultrasmall superparamagnetic iron oxide [274]. A novel AnxA5-conjugated nano-sized ultrasound contrast agent was able to detect apoptotic cells inside tumours after chemotherapy [275] (Table 5e). Magnetic beads coated with AnxA5 were recently used to isolate tumour-derived extracellular vesicles from mouse models xenografted with human cancer cells, an approach with diagnostic and therapeutic potential [276]. Another alternative to ^99m^Tc-AnxA5, which has limitations for non-invasive molecular imaging in the clinic [277], is ^68^Ga-labeled AnxA5, which efficiently visualized hepatic apoptosis in an anti-Fas antibody mouse model and efficacy of established chemo- and radiotherapies in animals with Burkitt’s lymphoma [278]. Alternatively, fusion of AnxA5 with a mutant of *Renilla* luciferase created a new and sensitive biosensor that effectively detected vascular apoptosis in ethanol-induced corneal apoptosis in mice [279]. Interestingly, labeling of AnxA5 with a pH-sensitive probe that emits fluorescence upon pH decrease provided opportunity to monitor efferocytosis, the clearing of apoptotic cells by phagocytosis [280] (Table 5f).

### 6.6. Other PS-Related Aspects of AnxA5 Biology

Other AnxA5-related biological activities reviewed in more detail elsewhere include viral infection, membrane invagination and membrane repair [11,226,281,282,283]. In addition, AnxA5-KO mice showed a strongly reduced allogeneic reaction against primary and secondary necrotic cells as well as carcinoma cells [284,285]. This could also be relevant for preventing viral infections, including human immunodeficiency virus, which together with infected monocytes/macrophages, exposes increased amounts of PS on the surface [284] (Table 5f). Possibly relevant in this context is the ability of the core domain of AnxA5 and several other annexins to interact with Dectin-1, which facilitates uptake of apoptotic cells by dendritic cells and is an important mechanism for establishing peripheral tolerance to self-antigens [286].

## 7. AnxA6

AnxA6 is highly and ubiquitously expressed, except in epithelial cells of the small intestine, colon and parathyroid gland [1,2,3,10,288]. AnxA6 is mainly found at the plasma membrane, endo- and exocytic vesicles, but also in mitochondria and lipid droplets [1,2,3,4,9,288,289,290]. AnxA6 consists of eight rather than four annexin repeats and interacts with a variety of proteins and lipids to regulate microdomain organization, endo- and exocytosis, cholesterol transport, signal complex (dis-) assembly, cytoskeleton rearrangements, and stress response. Hence, AnxA6 has been associated with cell growth and motility, differentiation, lipid and glucose homeostasis [1,2,3,4,9,10,288,291,292], as well as membrane repair [293,294,295] and viral infection [296,297,298].

Alternative splicing generates two isoforms, AnxA6-1 and AnxA6-2, the latter lacking six amino acids at the C-terminus (pos. 524–529) [288,299]. The AnxA6-1 isoform is abundant in most cells and tissues, while AnxA6-2 is upregulated in some transformed cell lines [288,300,301] and isoform-specific functions related to Ca^2+^ homeostasis and endo-/exocytosis may exist [288,302,303,304], but in vivo evidence supporting these observations is still lacking. Interestingly, computational models suggested limited overlap, but rather a diversity of functions associated with the two AnxA6 isoforms [305].

### 7.1. AnxA6 and Cardiac Function

Initial studies with mice overexpressing AnxA6 in the heart led to enlarged dilated hearts, acute diffuse myocarditis, lymphocyte infiltration and fibrosis throughout the heart and pulmonary veins, ultimately causing heart failure [306] (Table 6a). Furthermore, cardiomyocytes from *Anxa6^−/−^* mice showed higher contractility and accelerated removal of diastolic Ca^2+^ from the cytoplasm [307]. Hence, AnxA6 scaffold and membrane organizer functions probably regulate Ca^2+^-dependent ion pumps and/or exchangers. In addition, Ca^2+^ signaling and stimulus–response coupling in the heart were altered in mice overexpressing a dominant-negative N-terminal AnxA6 mutant [308]. As other N-terminal AnxA6 deletion mutants compromised endocytosis and signaling [309,310,311,312,313], these mutants probably lack Ca^2+^-dependent membrane-binding properties and remain cytosolic, trapping their interaction partners in the cytosol (Table 6a).

Nevertheless, AnxA6 deficiency in mice was not associated with alterations in the heart rate, blood pressure, cardiovascular response to septic shock or changes in B or T cell development [314] (Table 6a), challenging proposed AnxA6 functions in the heart and in lymphocyte differentiation [306,307,308,315].

### 7.2. AnxA6 and Skeletal Development

AnxA6 is highly expressed in bone and skeletal muscle, yet *Anxa6^−/−^* animals and even double KO-mice lacking AnxA6 and AnxA5 did not reveal defects in skeletal development [238,239] (see also Section 6.1). However, in later studies, a reduction in the growth plate length and number of chondrocytes in newborn AnxA6 KO-mice was observed, which correlated with reduced mineralization of growth plate cartilage [316]. In addition, *Anxa6^−/−^* chondrocytes exhibited delayed terminal differentiation, markedly decreased intracellular Ca^2+^ levels upon ascorbic acid stimulation and reduced PKCα membrane translocation and activity, the latter possibly decreasing MAPK signaling required for chondrocyte differentiation [316] (Table 6b).

In studies mimicking osteoarthritis, knee cartilage destruction after IL-1β injection or partial meniscectomy was strongly reduced in AnxA6 KO-mice [317]. Mechanistically, the loss of AnxA6 interacting and promoting nuclear translocation of NFκB, a major driver in the pathogenesis of osteoarthritis, could be responsible for this phenotype. Furthermore, in articular chondrocytes from control animals, AnxA6 associates with the plasma membrane to interfere with Wnt/b-catenin signaling, affecting crosstalk with NFκB signaling, catabolic and inflammatory events, all of which contributing to cartilage degradation in osteoarthritis [318].

The ability of AnxA6 to influence the organization of cholesterol-rich plasma membrane microdomains [1,2,3,4,288,319] on neuronal cells also appears relevant in osteoarthritis. AnxA6 KO-mice displayed increased sensitivity to mechanical stimuli due to elevated activity of the cation channel Piezo2 in sensory neurons, which mediates mechano-gated current linked to body awareness and touch [320]. Moreover, AnxA6 overexpression attenuated mechanical pain, indicating therapeutic potential to inhibit chronic mechanical pain commonly associated with osteoarthritis (Table 6b).

### 7.3. AnxA6 and Immune Response

Although AnxA6 is upregulated during B and T lymphocyte differentiation [315], unchallenged *Anxa6^−/−^* mice exhibited a normal B and T cell development [314], including a normal repertoire of CD3^+^, CD4^+^, CD8^+^, naïve (CD62L^+^CD44^−^), effector (CD62L^−^CD44^+^) and memory (CD62L^+^CD44^+^) T lymphocytes [321]. However, after triggering a delayed-type contact hypersensitivity reaction upon dermal exposure to an irritant, the local tissue swelling and clonal expansion of T cells in the lymph nodes from AnxA6 KO-mice contained significantly less proliferating CD4^+^ T lymphocytes [321] (Table 6c). Furthermore, IL-2 signaling, critical to elicit T cell proliferation, was impaired in AnxA6-deficient T cells [321]. This correlated with reduced plasma membrane order of AnxA6^−/−^ T cells, suggesting an improper formation of specialized (cholesterol-rich) membrane microdomains (lipid rafts), interfering with appropriate IL-2 receptor signaling during immune response [288,321,322].

Along these lines, membrane order and protein distribution in rafts and non-rafts was also altered in mesenchymal mouse embryo *Anxa6^−/−^* fibroblasts [319] (Table 6d). These membrane-organizing functions of AnxA6 might also affect other immune responses, as AnxA6-deficient mice showed reduced bacterial clearance after oral challenge with *Citrobacter rodentium*, indicating a higher susceptibility to infection (The Wellcome Trust Sanger Institute Mouse Genetics Project, Database Release 2011; http://www.informatics.jax.org/reference/J:175295; accessed 12 March 2021).

### 7.4. AnxA6 and Lipid and Glucose Homeostasis

AnxA6 has been linked to lipoprotein and cholesterol uptake and transport, cytokine secretion as well as signaling events related to lipid and glucose homeostasis [4,9,288,292], which appear relevant in liver and adipose physiology (Table 6e).

AnxA6 was found associated with lipid droplets in adipocytes [323,324] and AnxA6 upregulation in 3T3 adipocytes lowered triglyceride storage and secretion of adiponectin, which exerts beneficial effects on glucose and lipid homeostasis [323,324]. *Vice versa*, serum adiponectin levels were increased in AnxA6-deficient mice. As AnxA6 levels increased in WAT of obese mice, this may contribute to impaired lipid storage and adiponectin release associated with obesity [324]. AnxA6 is also associated with hepatic lipid droplets [290,325], with primary *Anxa6^−/−^* hepatocytes and liver sections of AnxA6 KO-mice showing reduced triglyceride accumulation and lipid droplet numbers [325]. As AnxA6 up- and downregulation occurs in hepatic lipid disorders, this may implicate a novel role for AnxA6 in fatty acid and triglyceride metabolism in the liver [325].

During the course of HFD-feeding, AnxA6 KO-mice gained less weight, in particular in WAT, compared to controls [326]. HFD-feeding caused elevated total plasma triglycerides and cholesterol levels, yet an overall lipoprotein profile commonly associated with increased insulin sensitivity was observed [326,327,328]. Indeed, AnxA6 deficiency resulted in increased hepatic insulin signaling and improved glucose clearance [326]. However, HFD-fed AnxA6 KO-mice failed to properly downregulate hepatic gluconeogenesis and revealed increased glycogen storage in the liver, implicating AnxA6 in the fine-tuning of hepatic glucose metabolism and systemic control of glucose [326] (Table 6e).

Strikingly, when the ability to regenerate the liver was challenged after partial hepatectomy (PHx), AnxA6-deficient mice exhibited a significantly lower survival rate after PHx [329]. This striking phenotype was not due to defects in hepatocyte cell cycle progression, or the delayed onset of steatosis in these animals, but a prolonged hypoglycaemia, which could not accommodate the heavy energy requirements in AnxA6^−/−^ animals after PHx. Mechanistically, shuttling of the Na^+^-coupled neutral amino acid transporter SNAT4 to the hepatocyte plasma membrane, which provides alanine as fuel to drive gluconeogenesis and fatty acid synthesis during liver regeneration, was compromised in *Anxa6^−/−^* mice [329]. Such a decisive role in the post-hepatectomy phase has yet been documented only for a rare number of genes [330,331,332].

The liver is central for cholesterol homeostasis and contains abundant amounts of AnxA6 [333], with potential roles in the uptake and internalization of LDL-derived cholesterol [309,310,334,335]. Moreover, AnxA6 overexpression induced cholesterol accumulation in late endosomes, a phenotype reminiscent of the Niemann-Pick type C1 (NPC1) mutation [336]. Outstandingly, AnxA6 depletion in NPC1 mutant cells alleviated cholesterol accummulation by restoring cholesterol export to the endoplasmic reticulum via Rab7 and StAR-related lipid transfer domain-3 (StARD3)-dependent formation of membrane contact sites [337]. In line with these findings, Rab7-GTP levels were elevated in mouse embryo fibroblasts from *Anxa6^−/−^* mice [337], indicating that the gatekeeper function of AnxA6 to control cholesterol transport from late endosomes could be relevant in vivo and the progression of NPC disease [338] (Table 6e).

In NPC disease, late endosomal cholesterol accumulation due to the loss of NPC1 primarily affects the brain, causing neuronal damage and affecting motor coordination. In addition, considerable liver malfunction is also common. Strikingly, lack of AnxA6 in NPC1-deficient animals (Npc1^−/−^/Anxa6^−/−^) did not prevent cerebellar degeneration [339]. Furthermore, livers of the AnxA6-deficient NPC1 KO-animals contained a significantly elevated number of foam cells congesting the sinusoidal space, which is commonly associated with inflammation, further deteriorating their compromised hepatic functions and reduced lifespan [339].

Lipid droplet numbers, cholesteryl esters and glycogen granules were strongly decreased in livers from *Npc1^−/−^/Anxa6^−/−^* mice, indicating a much higher turnover of cholesteryl esters, other neutral lipids and glycogen compared to NPC1 KO-animals [339]. In addition, and reflecting observations in liver, retina and primary fibroblasts from *Anxa6^−/−^* mice [289], morphologically altered mitochondria in *Anxa6^−/−^* hepatocytes were apparent [339]. In agreement with AnxA6 depletion rescuing the NPC1 mutant phenotype [337,338], AnxA6 deficiency slightly improved compromised mitochondrial morphology [340,341] and possibly inter-organelle communication in *Npc1^−/−^* animals [339] (Table 6e).

### 7.5. AnxA6 and Cancer

Numerous studies identified AnxA6 to exhibit tumour suppressing as well as tumour promoting activities, depending on the cancer cell type and degree of malignancy [311,342,343]. These opposing activities probably relate to the multiple and diverse AnxA6 scaffolding functions, which are likely to differ in the various cancer cells due to the different repertoire and availability of interaction partners. Besides data from cell culture models or expression analysis of patient cohorts, several studies in recent years examined roles for AnxA6 in tumour growth and progression in vivo (Table 6f).

In EGFR overexpressing A431 epithelial carcinoma lacking endogenous AnxA6 [311,312], restoration of AnxA6 expression promoted PKCα-mediated threonine 654-EGFR phosphorylation, which inhibited EGFR tyrosine kinase activity, cell growth, migration and invasion [313,344]. AnxA6 overexpression in these A431 cells also reduced growth and EGFR activity in A431 xenografts in a PKCα-dependent manner [345,346]. Moreover, AnxA6 up- or downregulation impacted on the efficacy of tyrosine kinase inhibitors to inhibit growth, migration and invasion of EGFR-related cancer cells [346].

In a TNBC model using BT-549 xenografts, AnxA6 depletion was associated with early onset and rapid growth of tumours, which correlated with poor overall survival of basal-like TNBC patients [347]. These studies identified RasGRF2, a Ras protein specific guanine nucleotide exchange factor (RasGEF), as an additional effector besides PKCa and p120 GTPase activating protein [3,8,311,312], to confer AnxA6-dependent roles in cell growth and motility [347]. In fact, the inverse relationship of AnxA6 and RasGRF2 expression levels discriminated rapidly growing from invasive TNBC subsets [348]. This could be relevant for subset-specific therapeutic interventions, as AnxA6 upregulation was associated with the response of TNBC cells to cytotoxic and/or EGFR-targeted therapies and the development of drug resistance [343,349] (Table 6f).

Significant amounts of AnxA6 are found in late endosomes/(pre-) lysosomes [9,288,292,335,336,337]. Inner membranes from this compartment can fuse with the plasma membrane to be released as extracellular vesicles (EVs) [4,292,350]. These EVs, including exosomes, are highly enriched in annexins, including AnxA6 (ExoCarta exosome database: www.exocarta.org; accessed 12 March 2021) and are believed to facilitate cell–cell communications and influence recipient cell behaviour.

In two mouse models of breast cancer (MMTV-PyMT, 4T1), taxane and anthracycline therapy induced secretion of EVs from tumours with enhanced metastatic capacity. These chemotherapy-elicited EVs were enriched with AnxA6 and promoted NFκB-dependent endothelial activation, cytokine secretion and monocyte subtype expansion in the pulmonary pre-metastatic niche to establish lung metastasis [351]. Hence, AnxA6 enrichment on EVs might be useful to develop a biomarker to predict the risk for metastasis in patients with poor response to chemotherapy. Similarly, AnxA6-positive EVs from CAFs supported PDAC aggressiveness, and was restricted to PDAC patients, making it a potential biomarker for PDAC grade [352]. Along these lines, AnxA6-containing EVs from CAFs also contributed to drug resistance in a peritoneal metastasis mouse model for gastric cancer, possibly via activation of integrin β1, focal adhesion kinase (FAK) and yes-associated protein 1 (YAP) signaling [353]. The identification of a monoclonal AnxA6 antibody with anti-invasive properties on aggressive pancreatic, lung squamous and breast cancer cells further supports pro-metastatic activity of extracellular AnxA6 [113] (Table 6f).

### 7.6. AnxA6 and Membrane Repair

Studies in human and rodent cell-based models implicated several annexins, including AnxA6, in membrane repair [11,287,294]. Likewise, in zebrafish, AnxA6 was rapidly recruited to damaged sarcolemma and cooperated with dysferlin to repair membrane lesions [293] (Table 6g). In this transparent and powerful model organism for the study of vertebrate biology, AnxA6 and other annexins accumulated at the site of muscle damage, with AnxA6 deficiency leading to myopathy [293]. Loss of dysferlin was also accompanied by impaired AnxA6 translocation and resealing after sarcolemmal disruption in mouse models [354]. Strikingly, in a mouse model of muscular dystrophy, a naturally occurring AnxA6 splice variant created a truncated AnxA6 protein lacking four of the eight annexin repeats. This mutant acted in a dominant-negative manner and interfered with membrane recruitment of full-length AnxA6 to substantially decrease efficiency of membrane repair [295]. Other genetic modifiers of muscular dystrophy also interfered with AnxA6 recruitment for sarcolemma resealing [355]. High resolution microscopy of muscle after laser injury revealed rapid recruitment of AnxA6 and other annexins (AnxA1, A2, A5) to damaged sarcolemma, contributing to the formation of a tight cap in a Ca^2+^- and actin-dependent manner over a vesicle-rich repair zone [11,287,295,356]. Interestingly, intermittent glucocorticoid treatment enhanced muscle repair, through increased expression of AnxA6 and AnxA1, without eliciting the well-known effect of steroids to trigger muscle atrophy [357]. Alternatively, administration of recombinant AnxA6 may protect against acute muscle injury, as it was capable to reseal injured membrane in mouse models [358] (Table 6g).

## 8. AnxA7

In contrast to all other annexins, AnxA7 contains a long (100 aa) hydrophobic N-terminus (Figure 1), with alternative splicing generating a 47 kD isoform found in all tissues except skeletal muscle, and a 51 kD isoform expressed in the heart, brain and myotubes [359]. AnxA7 is predominantly associated with secretory vesicles, the plasma membrane and the nuclear envelope in a Ca^2+^-dependent manner [360]. Many reports support AnxA7 to have GTPase activity and contribute to the regulation of Ca^2+^ homeostasis, exocytic pathways, and prostaglandin production, with consequences for pancreatic and cardiac functioning and inflammatory myopathies [361,362,363,364,365,366,367], but also cell survival and tumour growth [368,369].

### 8.1. AnxA7 and Pancreatic β-Cell Function

The first study examining the targeted disruption of the AnxA7 gene reported an embryonic lethality due to cerebral haemorrhage in homozygous AnxA7^−/−^ mice [361]. Yet, heterozygous *Anxa7^+/−^* mice of this strain were viable and the pancreas of these animals exhibited islet hyperplasia, β-cell hypertrophy, aberrant metabolic gene expression patterns and reduced inositol 1,4,5-trisphosphate receptor levels. The latter was considered responsible for defects in the Ca^2+^ release from intracellular stores, compromising Ca^2+^-dependent insulin secretion. In follow-up studies, altered ryanodine receptor (RyR) -mediated Ca^2+^ release was also related to abnormal insulin secretion from β-cells of these *Anxa7^+/−^* mice [370]. In contrast, an independently generated second AnxA7 KO-strain was viable and lacked any defect in Ca^2+^-induced and cAMP-mediated insulin secretion [371]. The different genetic background, design and integration sites of the targeting construct, orientation of the inserted *neo* gene and possible consequences for neighbouring genes could possibly explain the strikingly different phenotypes of the two *Anxa7^−/−^* strains [371] (Table 7a).

### 8.2. AnxA7 and Cardiac Function

AnxA7 is highly expressed in the heart and based on its possible role in Ca^2+^ homeostasis, adult cardiomyocytes from the viable AnxA7 KO-strain were analyzed [371]. These studies identified the misfunctioning of the apparatus responsible for cardiac muscle contraction, most likely due to defects in Ca^2+^ homeostasis [371]. In fact, AnxA7 interacts with Sorcin and RyR, which both couple Ca^2+^ channels to the contractile machinery in cardiac muscle [372], indicating that the loss of these interactions might contribute to the cardiac phenotype in *Anxa7^−/−^* animals [371,373].

In addition, the viable *Anxa7^−/−^* strain showed increased susceptibility to atrial fibrillation and ventricular tachycardia, both electrophysiological properties linked to the development of heart arrhythmia [374]. Furthermore, in atrial and ventral tissues of the *Anxa7^−/−^* mice, impaired integrity of the basement membrane of cardiomyocytes and lack of collagen fibers in intracellular spaces was observed, possibly contributing to the de-regulated conductive properties of these animals [374]. Moreover, when examining the consequences of increased resistance to blood flow out of the heart upon transverse aortic constriction, a well-established surgical procedure to induce cardiac hypertrophy, the increase in the ratio of heart to body weight was more pronounced in AnxA7 KO-mice. AnxA7-dependent cardiac activity of the transcription factor NFAT (nuclear factor of activated T cells), regulating genes involved in the hypertrophic response following cardiac stress conditions, could contribute to the observed phenotype [367] (Table 7a).

### 8.3. AnxA7 and Eryptosis

Besides cardiac complications, red blood cells from *Anxa7^−/−^* mice were characterized by an altered cell shape and increased resistance to osmotic shock [375]. Inspection of eryptosis, the suicidal death of erythrocytes, uncovered hyperosmotic shock, Cl^-^ removal or energy depletion to trigger enhanced COX-mediated prostaglandin E_2_ (PGE_2_) production in *Anxa7^−/−^* erythrocytes [376]. This elicited cytosolic Ca^2+^ elevation, subsequent PS exposure at the cell surface, and ultimately, apoptotic death. Eryptosis was also accelerated in *Plasmodium*-infected *Anxa7^−/−^* erythrocytes, indicating that AnxA7 downregulation may partially protect against malaria in vivo [377]. Although several annexins have previously been proposed to inhibit PLA_2_ [1,2,10], which acts immediately upstream of COX in the prostaglandin synthesis pathway, only pharmacological inhibition of COX, but not PLA_2_, abolished the differences in parasitemia and erythrocyte survival between *Anxa7^−/−^* and wildtype animals [377] (Table 7a).

Further supporting an inhibitory role for AnxA7 in PGE_2_ production, glucocorticoid-induced gastric acid secretion, which requires simultaneous downregulation of COX-mediated PGE_2_ production, was compromised in *Anxa7^−/−^* mice [378].

Additionally, plasma prostaglandin levels and hepatic COX activity were significantly elevated in AnxA7 KO-mice [366]. As PGE_2_ upregulation can induce insulin resistance in hepatocytes, this might contribute to the decreased glucose tolerance observed in these animals. Pharmacological COX inhibition, using aspirin, improved glucose tolerance in the AnxA7-deficient strain, but also abrogated differences in insulin levels in wildtype and AnxA7 KO-animals (Table 7a).

In other studies, the increased PS exposure and eryptosis of *Anxa7^−/−^* erythrocytes were associated with an increased adhesion to endothelial cells, an observation that might be relevant for the adhesion of erythrocytes to the vascular wall and contribute to impaired microcirculation during acute ischemic renal failure [379].

Enhanced PGE_2_ formation upon AnxA7 depletion appears relevant also for implantation and fertility [380]. In women suffering from recurrent pregnancy loss, decreased AnxA7 levels in endometrial biopsies during the midluteal window of implantation were observed. This correlated with the viable *Anxa7^−/−^* strain showing significantly increased implantation sites and litter sizes, indicating that ANXA7-mediated regulation of COX-dependent PGE_2_ production contributes to regulating endometrial receptivity and implantation [380].

### 8.4. AnxA7 and Brain Injury

Several studies addressed roles for AnxA7 in the brain (Table 7a). Possibly linked to AnxA7-related impacts on the cellular handling of Ca^2+^ (Section 8.1, Section 8.2 and Section 8.3), lack of AnxA7 affected Ca^2+^ homeostasis and proliferation of primary *Anxa7^−/−^* astrocytes [381].

Other research suggested PKC-mediated phosphorylation of ANXA7 to promote interaction with the SNARE proteins SNAP23 and SNAP25, facilitating membrane fusion of exocytic vesicles with the plasma membrane [363,382]. These regulatory circuits may be relevant for presynaptic glutamate release and postsynaptic N-methyl-D-aspartate receptor trafficking, as proposed in studies examining brain tissue around hematoma after intracerebral hemorrhage-induced brain injury [382]. In this model, ANXA7 knockdown in vivo showed rescue effects on neuronal death, blood–brain barrier damage, brain edema, neurobehavioral deficient, and inflammatory response. Similar results were also obtained after subarachnoid hemorrhage-induced brain injury [383] and correlate with elevated AnxA7 levels in traumatic brain injury [384], making AnxA7-based therapies a potentially novel and alternative approach in the treatment of cerebrovascular disease (Table 7a).

### 8.5. Small Molecules Targeting AnxA7

Given the therapeutic potential of AnxA7 in a variety of disease settings, efforts to screen small molecules identified 6-amino-2, 3-dihydro-3-hydroxymethyl-1, 4-benzoxazine (ABO) to selectively bind and interfere with AnxA7 phosphorylation, altering AnxA7 localization, expression levels and its GTPase activity [385]. ABO was initially found to induce AnxA7-dependent autophagy in cell-based studies [385]. In apoE^−/−^ mice fed a Western diet and treated with ABO, elevated AnxA7 levels in the aortic endothelium correlated with increased autophagy and reduced apoptosis in this location. Moreover, this was associated with reduced atherosclerotic plaque size and a more stable plaque phenotype, characterized by reduced lipid deposition, improved ratio of pro-/anti-inflammatory macrophages, increased collagen and smooth muscle cell content, and less apoptosis [386]. The underlying mechanisms how ABO-mediated inhibition of AnxA7-GTPase activity could reduce the size and stability of atherosclerotic plaques are complex, and include altered expression of proteins involved in survival, metabolism, and autophagy [387,388], respectively (Table 7a).

### 8.6. AnxA7 and Cancer

In human cancers, deletions at the *Anxa7* gene locus on chromosome 10q21 are common, and a substantial number of studies identified AnxA7 and its GTPase activity as a tumour suppressor with roles in cell death, motility and invasion in a variety of cancers [368,369]. For instance, ANXA7 expression is commonly lost in hormone-refractory and locally recurrent metastatic prostate cancer, glioblastoma, and melanoma. On the other hand, AnxA7 upregulation and tumour-promoting roles in the initiation and progression of liver, colorectal, gastric, nasopharyngeal and breast cancer, have been reported. It would go beyond the scope of this review to recapitulate these opposing findings in more detail, but often the underlying mechanisms can be associated with alterations in Ca^2+^ homeostasis, Ca^2+^/GTP-dependent exocytic events, expression patterns of downstream effectors, as well as AnxA7-dependent protein interactions [368,369].

Several in vivo studies support tumour suppressor roles for AnxA7 (Table 7b). For instance, the initially generated heterozygous *Anxa7*^+/−^ strain [361] revealed a cancer-prone phenotype, with more than 20% of these mice developing spontaneous neoplasms, with lymphosarcoma being most frequent, especially in females, indicating gender- and possibly sex hormone -related differences [389]. This was accompanied by reduced expression of several tumour suppressors, DNA repair- and apoptosis-related genes, indicating the development of genomic instability driving disease progression upon partial loss of AnxA7 [389].

In efforts to develop therapeutic strategies against cancer cells expressing high integrin β4 levels, which is common in multiple carcinomas, a small molecule ((S)-ethyl 1-(3-(4-chlorophenoxy)-2-hydroxypropyl)-3- (4 methoxyphenyl)-1H-pyrazole-5-carboxylate) (SEC) that increased AnxA7 GTPase activity similar to ABO was identified [390]. In these studies, SEC-induced AnxA7 bound and promoted the nuclear translocation of integrin β4, which inhibited the growth of A549 xenograft tumours in the avian embryo model [390]. Likewise, SEC-mediated activation of AnxA7 GTPase effectively inhibited prostate cancer metastasis in vivo [391]. In the latter, AnxA7-mediated activation of 5’ AMP-activated protein kinase (AMPK) suppressed mammalian target of rapamycin complex 1 (mTORC1)/STAT3 signaling responsible for the expression of pro-metastatic genes [391].

On the other hand, AnxA7 upregulation was proposed to increase tumourgenicity and lymph node metastasis in a hepatocarcinoma mouse model [392]. Vice versa, miR124-3p-mediated downregulation of AnxA7 inhibited hepatocellular carcinoma growth, invasion and lymphatic metastasis [393]. Silencing AnxA7 in a xenograft model for gastric cancer induced apoptosis, altered the expression of apoptosis-mediating genes, and inhibited tumour growth [394]. Altogether, AnxA7 has potential as a therapeutic target, but may also serve as a biomarker for the diagnosis, treatment and prognosis of a number of tumours [368,369] (Table 7b).

## 9. AnxA8

AnxA8 is a less well-characterized member of the annexin family, and expressed at low levels in the lung, liver, kidney, skin, placenta, and the cornea [395,396]. AnxA8 levels are upregulated by leptin, during mammary gland involution and in acute promyelocytic leukemia (APL) cells [396,397,398]. In these APL cells, all-*trans* retinoic acid-induced differentiation was associated with AnxA8 downregulation, indicating a role in proliferation and differentiation [398]. Indeed, over the years, elevated AnxA8 levels were reported in a variety of cancers [397,398,399], and as shown upon in vivo knockdown in endometrial cells of the uterine horn of mice, possibly promoting cell proliferation via Akt signaling pathways [400] (Table 8).

AnxA8 controls the sorting and transport of late endosomal cargo [401], a function that appears critical for leucocyte adhesion to activated endothelium, which requires stabilization of the leukocyte receptor P-selectin on the endothelial cell surface by CD63. CD63 is located in late endosomes and in order to fulfill this function, needs to be transported to the cell surface. Strikingly, cell surface delivery of CD63 in AnxA8-deficient human umbilical vein endothelial cells was strongly reduced and correlated with compromised leukocyte adhesiveness in inflammatory-activated endothelial venules of AnxA8-deficient mice [402] (Table 8).

## 10. AnxA9

AnxA9 was initially identified in expression libraries from fetal liver/spleen [403] and shares a high sequence homology with AnxA2. Yet, AnxA9 is an atypical annexin, as inactivated type II-Ca^2+^ binding sites within its core domain confered Ca^2+^-independent membrane and phospholipid-binding properties [404]. Together with its low abundance during mouse development and in adult organs, in particular spleen and liver [405], this suggests a Ca^2+^-independent and rather specific role unrelated to membrane scaffolding functions proposed for the other annexins. Interestingly, autoantibodies isolated from patients with pemphigus vulgaris, a rare skin autoimmune disease, cross-reacted with AnxA9 [406]. However, the exact role of AnxA9 in this disease has yet to be clarified and an AnxA9 KO-mouse model does not exist.

## 11. AnxA10

Annexin A10 was identified only two decades ago [407] and is mainly expressed in epithelia of the gastrointestinal tract [408]. Its localization and function, alike AnxA8, remains to be fully understood, but unlike other annexins, AnxA10 displays functions independent of Ca^2+^ and/or membrane-binding and was found in nuclear bodies interacting with mRNA-binding proteins, possibly involved in mRNA regulation or processing [409]. Homozygous deletion or downregulation of AnxA10 in several cancers, including bladder, gastric, pancreas and liver, correlated with progression and poor survival, indicating potential as a diagnostic marker and tumour suppressor [408,410,411,412].

To our knowledge, up to date, only two studies utilized gene depletion approaches to examine AnxA10 functions in vivo, both addressing neuropathic pain. In mice, spinal nerve ligation (SNL) -induced neuropathic pain was associated with AnxA10 upregulation in the spinal cord. AnxA10 colocalized with neuronal and astrocyte markers and upon AnxA10 depletion, SNL-induced pain was attenuated [413]. Similarly, AnxA10 knockdown in the spinal cord of rats suppressed SNL-induced hyperalgesia, possibly by blocking the activation of pro-inflammatory mediators, including NFκB, TNFα and IL-1β and metalloproteases [414] (Table 9).

## 12. AnxA11

AnxA11 is ubiquitously expressed in a wide variety of tissues with diverse functions in cytoplasmic and nuclear locations. Earlier studies identified AnxA11 to associate with the nuclear envelope in a Ca^2+^- and cell cycle-dependent manner, binding to S100A6 (calcyclin) [415], an interaction possibly relevant for cell growth and differentiation [415,416,417]. During cell cycle progression, AnxA11 translocates from the nucleus to the spindle poles in metaphase and to the spindle midzone in anaphase, enabling midbody formation and completion of cytokinesis [417]. In the cytosol, AnxA11 binds to secretory vesicles in a Ca^2+^-dependent manner, regulating Ca^2+^-dependent exocytic events, such as Ca^2+^-inducible insulin secretion in pancreatic β-cells [418]. In the early secretory pathway, interaction of AnxA11 with apoptosis-linked gene-2 (Alg-2) and Sec31A contributes to the exit of cargo from the endoplasmic reticulum for transport to the Golgi apparatus [419].

### 12.1. AnxA11 and Cancer

Although confirmation from in vivo studies in mouse models are still limited, in certain cancers AnxA11 up- or downregulation appears to be linked to disease progression and development of drug resistance [93] (Table 10). For instance, in ovarian cancer AnxA11 downregulation correlated with tumour recurrence and was associated with cisplatin resistance [420]. In a mouse model for human hepatocellular carcinoma, ANXA11 upregulation was associated with increased Akt activation and contributed to tumour growth and progression [421]. Species and/or cancer-subtype specific differences may exist, as ANXA11 downregulation in the murine Hca-P hepatocarcinoma cell line promoted in vivo tumour growth, lymph node metastatic potential and chemoresistance towards 5-fluorouracil [422].

Strikingly, a single nucleotide polymorphism (SNP) in the *Anxa11* gene, causing a mutation at position 230 (R230C), was associated with an increase in the overall response rate of metastatic colorectal cancer patients to bevacizumab, an angiogenesis inhibitor [423]. In support of these findings, the R230C variant was more susceptible to bevacizumab-induced tumour suppression when examining xenograft models [424]. The underlying mechanism remains to be clarified, but this *Anxa11* SNP (rs1049550) may serve to improve the identification of metastatic colorectal cancer patients sensitive to bevacizumab regimens [425].

### 12.2. AnxA11 and Autoimmune Diseases

Besides the link of several annexins, including AnxA11, to the autoimmune disease SLE, *Anxa11* polymorphism were found in other autoimmune disorders, such as granulomatous disease [426]. Furthermore, several genome-wide studies in recent years identified a genetic association of the R230C variant with sarcoidosis [427,428].

Finally, *Anxa11* mutations were found in the rare and related neurodegenerative diseases amyotrophic lateral sclerosis (ALS) and frontotemporal dementia. Numerous mutations in both the N-terminal tail and the C-terminal membrane binding region of *Anxa11* have now been described and may account for up to 6% of familial ALS in Chinese populations [429,430,431,432].

Recent studies provided mechanistic insight, proposing disease-associated *Anxa11* mutations to contribute to ALS pathogenesis through dysregulated Ca^2+^ homeostasis and abnormal protein aggregation [433]. Furthermore, *Anxa11* mutations in ALS altered several fundamental AnxA11 properties. In primary rat neurons, but also in an in vivo zebrafish model, ANXA11 acted as a molecular tether, coupling RNA granules to lysosomes, enabling long-distance transport of RNA along axons [432]. This novel relationship between lysosomes and RNA was disrupted in ALS-associated *Anxa11* mutations, as RNA granules could not dock onto lysosomes for axonal transport. Hence, *Anxa11* mutations may reduce delivery of essential mRNAs to distal regions of the neuron, which may dysregulate neuronal homeostasis and synaptic activity [432].

## 13. Conclusions

Exposure of annexin KO-mice or animals harbouring implanted annexin-deficient cell lines to a variety of disease-related stress conditions revealed important biological functions of individual annexins (Table 1, Table 2, Table 3, Table 4, Table 5, Table 6, Table 7, Table 8, Table 9 and Table 10). These animal models enabled the characterization of prominent extracellular activities for several annexins (i.e., AnxA1, A2, A5). This group of annexins might in the future serve as diagnostic or therapeutic tools—maybe in combination with nanoparticles or radio- and fluorescence-labeling. Peptide or protein-based approaches could be suitable to facilitate targeted delivery in vivo. AnxA1 and its interaction with FPR receptors has potentially numerous therapeutic applications in the context of acute and chronic inflammation and cancer (Section 2.1, Section 2.2, Section 2.3 and Section 2.4). The extracellular functions of AnxA2 are therapeutically relevant to control fibrinolysis and neoangiogenesis in several diseases (Section 3.1 and Section 3.3). The strong affinity of AnxA5 for PS on apoptotic cells can be applied in a large range of diagnostic and therapeutic approaches (Section 6.2, Section 6.3, Section 6.4, Section 6.5 and Section 6.6).

Phenotypes associated with the annexins AnxA3, A4, A6, A7, A8, A10 and A11 are often linked to Ca^2+^ homeostasis, as well as their membrane organizing and scaffolding properties, affecting receptors, transporters, or ion channels and their communication with cellular protein networks. However, the multifunctionality of these annexins render the development of targeted approaches difficult and fine-tuned local modulation of specific protein-protein or protein-lipid interactions remain hard to achieve. Nevertheless, drug targeting the GTPase activity of AnxA7 via small molecules hold promise for treatment of certain cancers and cardiovascular diseases (Section 8.5 and Section 8.6). In addition, drug-induced AnxA6 upregulation, delivery of recombinant AnxA6 or AnxA6-targeting antibodies might lead to the development of novel treatment options in lipid disorders, liver dysfunction, several cancers and muscular damage (Section 7.4, Section 7.5 and Section 7.6). Finally, the controlled up- or downregulation of annexins using viral gene delivery, small pharmacological molecules or CRISPR/Cas technology could provide opportunities for therapy in certain organs, such as liver and muscle, but also in the context of many cancer-related settings. Their intra- and extracellular expression levels could have diagnostic value, possibly linked to cancer progression and/or anticancer drug resistance (Section 3.3, Section 4.1, Section 5.1, Section 6.4 and Section 7.5).

On the other hand, the actual genetic evidence to support the relevance of annexins in human disease is still sparse. In earlier studies, annexin anomalities, also called ‘annexinopathies’, were initially reported in two disease states, identifying AnxA2 overexpression in patients with a haemorrhagic form of acute promyelocytic leukaemia, and AnxA5 downregulation on placental trophoblasts in the antiphospholipid syndrome [434,435]. Over recent years, a substantial number of additional studies identified correlations between disease progression and expression patterns of individual annexins; as is the case in many cancer and other acute and chronic diseases [1,2,3,21,22,23,90,116,226,242,342,343,368,369]. However, aided by deep sequencing data, various SNPs were identified, suggesting a potential involvement of AnxA1 in Alzheimer’s disease [436], and revealed roles for AnxA2 in sickle cell disease [119,120], but also cardiovascular risk due to elevated LDL cholesterol [114,149]. Similarly, the identification of SNPs in the *Anxa3* gene locus may imply roles in gastric cancer [437]. SNPs in the *Anxa5* gene are associated with the risk of pregnancy-related venous thrombosis, preeclampsia [438,439] and malignant melanoma [440]. Certain SNPs in the *Anxa6* locus have been linked to psoriasis [441,442], while another polymorphism in the *Anxa6* coding region causes a dominant-negative deletion mutant in a mouse model for muscle dystrophy that interferes with membrane repair [295].

Finally, and most convincingly, the R230C variant and several other SNPs in the N- and C-terminal region of *Anxa11* have been associated with neurodegenerative and autoimmune disorders, such as ALS [429,430,431,432,433] and sarcoidosis [427,428,429]. These polymorphism could be explored further and might serve as a marker for anticancer drug performance in metastatic colorectal cancer [423,424,425]. These observations, together with advanced intravital imaging techniques, –omics approaches, molecular modelling and small molecule development, in combination with tissue specific knock-in/KO-mouse models could open exciting perspectives to further pursue annexin-based therapeutic strategies.

## Figures and Tables

**Table 1:** Phenotypes observed in AnxA1 in vivo models.

**(a) AnxA1 and Acute/Chronic Inflammation**				
**Disease**	**Model**	**Phenotype**	**Mechanism**	**Ref**
Paw oedema, peritonitis, non-alcoholic steatohepatitis, rheumatoid arthritis, multiple sclerosis, colitis, asthma, lung fibrosis, bacterial infection, sepsis, allergic conjunctivitis, gout inflammation	AnxA1 KO-mice; FPR KO-models; Administration of recombinant AnxA1 or AnxA1 peptides (Ac2-26, CR-AnxA1_2-48_)	Increased (glucocorticoid-dependent) activation of innate immune response: neutrophil and monocyte recruitment, clearance of apoptotic cells, mast cell activation, reduced inflammatory cytokines	AnxA1 binds to FPR2: downstream effectors incl. MAPK, Akt, JNK, Ca^2+^ homeostasis, chemokine receptors, transcription factors, inflammatory mediators	[24,28,29,30,31,37,38,39,40,41,42,43,44,55,56,57,58,59,60,61,62,63,64,65,66,67,68,69,70,81] reviewed in [21,22,23,25,26,27,33,34,35]
**(b) AnxA1 and Cardiovascular Disease**				
Stroke	I/R injury or intracerebral hemorrage; Administration of recombinant AnxA1, Ac2-26 or CR-AnxA1_2-48_	AnxA1 counteracts pro-inflammatory response; Delayed or regressed progression of tissue damage	AnxA1/FPR2 and downstream effectors	[48,49,50,81] reviewed in [22,23,45]
Atherosclerosis	HFD-fed LDLR-KO, AnxA1/ApoE Double-KO; Administration of recombinant AnxA1 or Ac2-26	Reduced lesion size and macrophage accumulation	AnxA1/FPR2 and downstream effectors	[32,51] reviewed in [22,23,45]
Restenosis	Mechanical wire injury in HFD-fed AnxA1/ApoE Double-KO	Aggravated neointima development, macrophage accumulation	AnxA1/FPR2 and downstream effectors	[52]
Myocardial infarction (MI)	AnxA1 KO-mice; Rescue with AnxA1 administration	Increased necrosis, inflammation, hypertrophy, fibrosis	AnxA1/FPR2 and downstream effectors	[53,54]
**(c) AnxA1 and Tissue/Membrane Repair**				
Muscle injury	Cardiotoxin-induced tibialis anterior injury in WT mice	Less inflammation; Restored muscle repair	AnxA1 delivery via neutrophil recruitment	[66]
Muscle injury	Notexin-induced injury in AnxA1 KO-mice	Normal myofiber repair; Reduced myofiber regeneration	Cell–cell fusion	[67]
Muscle injury	Myofibers of ANO5 KO-mice	Reduced AnxA1 accumulation at injury site for cap formation	Membrane resealing	[113]
Skin grafting	Ac2-26 administration in WT mice	Improved skin transplantation and angiogenesis	AnxA1/FPR2 and downstream effectors	[60]
Mechanical injury-induced corneal scarring	Ac2-26 administration in WT mice	Improved corneal epithelial wound healing	AnxA1/FPR2 and downstream effectors	[68]
Intestinal wound repair	AnxA1 KO-mice; Rescue with AnxA1 administration	Defects in intestinal mucosal wound repair and epithelial cell migration	AnxA1/FPR2 and downstream effectors	[57]
Skin wound healing	AnxA1 KO-mice	Unchanged wound closure, inflammation, granulation tissue formation	AnxA1 dispensible in wound closure?	[80]
**(d) AnxA1 and Central Nervous System**					
Epilepsy	Ac2-26 administration in pilocarpine-induced status epilepticus	Anti-inflammatory and neuroprotective	AnxA1/FPR2 and downstream effectors	[71]
Epilepsy	Ac2-26 administration in kainic acid-induced temporal lobe epilepsy	Anti-inflammatory and neuroprotective	AnxA1/FPR2 and downstream effectors	[72]
Sepsis	Ac2-26 administration in cerebral inflammation	Anti-inflammatory and neuroprotective	AnxA1/FPR2 and downstream effectors	[73]
Alzheimer’s disease	Ac2-26 administration in Alzheimer’s mouse model	No improvement	Ac2-26 not crossing blood–brain barrier?	[74]
**(e) AnxA1 and Diabetes**				
Type 2 diabetes	HFD-induced insulin resistance in AnxA1 KO-mice	Females more susceptible to HFD-induced weight gain and insulin resistance	AnxA1/FPR2 and downstream effectors	[59]
Type 2 diabetes	HFD-induced insulin resistance in AnxA1 KO-mice; Ac2-26 administration in HFD-fed WT mice	More diabetic phenotype in AnxA1 KO-mice; Ac2-26 attenuated insulin resistance in WT	AnxA1/FPR2 and downstream effectors	[34]
Type 1 diabetes	Ac2-26 administration in streptozotocin (STZ)-treated AnxA1 KO-mice	Reduced cardiac and renal defects	Reduced FPR2 signaling	[75]
Type 1 and Type 2 diabetes	STZ-treated and HFD-fed AnxA1 KO-mice	Worse pathological remodelling of vasculature in insulin-resistant (HFD) mice		[76]
Type 1 diabetes	Compound 17b administration in STZ-treated mice	Vasoprotection; Reversal of endothelial dysfunction	Upregulation of vasodilating prostanoids	[77]
Type 1 diabetes	Transplantation of AnxA1-pretreated islets in STZ-mice	Restoration of glucose-stimulated insulin secretion	AnxA1/FPR2 and Ca^2+^ signaling	[47,78]
Type 2 diabetes	Ac2-26 administration during wound healing in diabetic db/db mice	Ac2-26 induced anti-inflammatory and pro-repair effects	AnxA1/FPR2	[79]
**(f) Nano-Based Delivery Approaches of AnxA1 Peptides**				
Atherosclerosis	Ac2-26 on nanoparticles targeting collagen IV in plaque	Localized Ac2-26 delivery; Reduced lesion instability	AnxA1/FPR2	[82]
Bowel disease	Oxidation-labile Ac2-26 containing nanoparticles to inflamed colons of mice	Reduced gastrointestinal inflammation, improved intestinal wound healing	AnxA1/FPR2	[83]
Arthritis	AnxA1-containing microvesicles from neutrophils	Resolving of cartilage protection in arthritic mice	AnxA1/FPR2	[84]
Cerebral ischemia	Fusion of AnxA1 with cell-penetrating peptide	Protection against neuronal apoptosis	Anti-apoptotic signaling?	[85]
Acute colitis	Grafting of small AnxA1 peptide into stable cyclic peptide scaffold	Reduced inflammation	AnxA1/FPR2	[86]
Breast cancer	Doxorubicin-DNA aptamer conjugate treatment of 4T1 tumours	Enhanced efficacy of targeted cancer therapy	Drug conjugate binds AnxA1 on cancer cell surface	[87]
Brain cancer	Peptide-mediated anticancer drug delivery (SN38) to B16 and C6 brain tumours	Effective suppression of brain tumour growth	Intravenously injected peptide-drug conjugate crosses blood–brain barrier and targets tumour vasculature via AnxA1	[88]
Systemic lupus erythematosus (SLE)	Monoclonal Anti-AnxA1 delivery in humanized SLE mouse model	Suppression of autoantibodies, lupus-associated cytokines and disease symptoms	Antibody-mediated AnxA1 inhibition downregulates autoreactive T and B cells	[89]
**(g) AnxA1 and Cancer**				
Fibrosarcoma, Non-small cell lung cancer (NSCLC)	AnxA1-depleted MCA205 and TC-1 xenografts	Anticancer immune response after chemotherapy	AnxA1/FPR1	[90,91,92]
Triple-negative breast cancer (TNBC)	4T1 tumours in AnxA1 KO-mice	Reduced cancer growth and macrophage infiltration of tumours	AnxA1/FPR2	[94]
Triple-negative breast cancer (TNBC)	Treatment of 4T1 tumours with AnxA1 antagonist	AnxA1 binding to FPR2 impedes anti-tumour immune response	AnxA1/FPR2	[95]
Breast cancer	MDA-MB-231 xenografts	Blocking AnxA1 binding to FPR1 reduces tumour growth/aggressiveness	AnxA1/FPR1	[96]
Breast cancer	Mammary gland epithelial cells derived from ANXA1 KO-mice	Up-/downregulation of signaling pathways in cell motility and cancer initiation	AnxA1/FPR2?	[97]
Pancreatic cancer	AnxA1-depleted, orthotopic MIA PaCa-2 xenografts	Less aggressive, reduced liver metastasis	AnxA1 pathways independent of FPRs	[100]
Multiple myeloma	AnxA1-depleted NCI-H929 tumours	Improved bortezomib treatment		[101]
Nasopharyngeal carcinoma	AnxA1-depleted CNE2 xenografts	Elevated radiotherapy resistance		[102]
**(h) AnxA1 and Intracellular Activities in Disease**				
Inflammation	Macrophages from AnxA1 KO-mice	Inflammatory cytokine production (IFN-β, TNFα), lipid mediators	Signaling pathways and NLRP3 inflammasome	[103,104,105,106]
Inflammation	Macrophages from AnxA1 KO-mice	Inflammatory cytokine production (IFN-β, TNFα), lipid mediators	Nuclear AnxA1 translocation; transcription, mRNA transport and stability	[103,104]
Prostanoids	*Anxa1^−/−^* fibroblasts	COX and cPLA_2_ upregulation	Glucocorticoid-independent	[24,108]
Membrane transport: Exocytosis	AnxA1^−/−^ mesenchymal stroma cells	Secretrome unable to stimulate insulin secretion from pancreatic b-cells	Exocytic membrane transport	[47,78]
Membrane transport: Late endosomes/lysosomes	*Anxa1^−/−^* fibroblasts	Biogenesis of internal vesicles in multivesicular bodies (MVBs)	Inward vesiculation	[109]
Membrane transport: Membrane contact sites (MCS)	*Anxa1^−/−^* fibroblasts	EGFR trafficking and cholesterol transfer	Establishment of ER/endosome contact sites	[110]
Membrane transport: Influenza infection	AnxA1 KO-mice	Reduced influenza infection	Reduced uptake and exit from MVBs	[111]
Membrane transport: Influenza infection	Influenza infection of lung cells from WT mice after AnxA1 administration	AnxA1 protects against viral infection	AnxA1/FPR2	[112]

Abbreviations: Akt, protein kinase B; ANO5, Anoctamin 5; Anx, annexin; ApoE, apolipoprotein E; COX, cyclooxygenase; cPLA_2_, cytoplasmic phospholipase A_2_; EGFR, epidermal growth factor receptor; ER, endoplasmic reticulum; FPR, formylated peptide receptor; I/R, ischemia/reperfusion; HFD, high-fat diet; IFN-β, interferon-β; JNK, janus kinase; KO, knockout; LDLR, low density lipoprotein receptor; MAPK, mitogen-activated protein kinase; MCS, membrane contact sites; MI, myocardial infarction; miR, micro RNA; MVBs, multivesicular bodies; SLE, systemic lupus erythematosus; STZ, streptozotocin; TNFα, tumor necrosis factor α; WT wildtype.

**Table 2:** Phenotypes observed in AnxA2 in vivo models.

**(a) AnxA2 and Vascular Homeostasis and Angiogenesis**				
**Disease**	**Model**	**Phenotype**	**Mechanism**	**Ref**
Acute carotic artery injury	AnxA2 KO-mice	Deposition of fibrin; Increased thrombosis	Src- and PKC-dependent AnxA2/p11-translocation and docking site for plasminogen and tPA	[122,123,124,125], reviewed in [115,116,117,119,120,121]
Diabetic retinopath	AnxA2 KO-mice	Compromised neoangiogenesis in matrigel implant, corneal pocket and oxygen-induced retinopathy	AnxA2/p11-mediated plasmin generation and vascular fibrinolysis	[114,121,126]
Stroke	AnxA2 administration in rat model of ischemic stroke	Prevention of stroke	Exogenous AnxA2 amplified tPA-mediated thrombolysis	[128]
Atherosclerosis	AnxA2/ApoE Double-KO mice	No changes in lesion development		[115]
Atherosclerosis	AnxA2/ApoE Double-KO mice	Reduced development of atherosclerosis in regions with disturbed flood flow	Suppressed integrin α5 signaling caused by oscillary shear stress	[129]
Vascular fibrinolysis	AnxA2 administration in EPAC KO-mice	Improved fibrinolytic function	AnxA2/p11-mediated plasmin generation and vascular fibrinolysis	[130]
**(b) AnxA2 and Pulmonary Vasculature**				
**Disease**	**Model**	**Phenotype**	**Mechanism**	**Ref**
Alveolar hypoxia	AnxA2 KO-mice	Reduced endothelial cell barrier function; Pulmonary endema, neutrophil infiltration in lung parenchyma	AnxA2 complex with endothelial cadherin and tyrosine phosphatases	[131]
Lung injury, idiopathic pulmonary fibrosis (IPF)	AnxA2 KO-mice	Reduced lung injury and IPF	AnxA2 augments factor Xa fibrogenic activity	[132]
Bleomycin-induced pulmonary inflammation and fibrosis	AnxA2 KO-mice; Administration of AnxA2 inhibitor (TM601) or AnxA2 antibodies	Reduced lung inflammation and fibrosis	AnxA2 binds bleomycin. This impedes TFEB-mediated autophagy to cause pulmonary fibrosis	[132,133,134]
Congenital muscular dystrophies and respiratory dysfunction	AnxA2 KO-mice	Similar to COL6 deficiency: reduced exercise tolerance and impaired lung tissue elasticity	Lack of COL6 in basement membrane, defect in SNARE-dependent secretory pathway	[135,136,137]
**(c) AnxA2 and Other Aspects of Vascular Homeostasis**				
**Disease**	**Model**	**Phenotype**	**Mechanism**	**Ref**
Cerebral venous thrombosis, antiphospholipid syndrome	AnxA2 KO-mice; Administration of recombinant AnxA2; patient data	Elevated AnxA2 autoantibodies lead to thrombotic complications	AnxA2/p11	[115,119,120,141,142]
Collagen-induced arthritis	Administration of recombinant AnxA2	Increased neovascularization and inflammation	AnxA2 binding to AnxA2 receptor induces Hedgehog signaling	[143,144]
Neovascular-related diseases	AnxA2 receptor overexpression in mouse aortic ring assays and mouse matrigel plug assay	Reduced Neovascularization	Suppression of KLF2 ubiquitin-dependent protein degradation	[145]
Cerebrovascular injury	AnxA2 KO-mice	Increased inflammation and reduced endothelial permeability/integrity (blood–brain barrier)	AnxA2/p11? AnxA2 receptor?	[146,147]
**(d) AnxA2 and Lipid Disorders**				
**Disease**	**Model**	**Phenotype**	**Mechanism**	**Ref**
Hypercholesterolemia, CVD	AnxA2 KO-mice; hepatic AnxA2 overexpression; patient data	Elevated LDL-cholesterol and PCSK9 levels	AnxA2 or AnxA2/p11 inhibits PCSK9-mediated LDLR downregulation	[114,148,149]
Diabetes, obesity, metabolic syndrome	AnxA2 depletion in obesity-induced insulin resistance	Attenuated insulin resistance	Suppression of NFκB signaling	[150]
Obesity	AnxA2 KO-mice	Reduced fatty acid uptake in WAT	AnxA2 complex with prohibitin and CD36	[151]
**(e) AnxA2 and Cancer**				
**Disease**	**Model**	**Phenotype**	**Mechanism**	**Ref**
Pancreatic ductal adenocarcinoma (PDAC)	AnxA2-KO crossed with PDAC mouse model	Reduced PDAC invasion and metastasis	Loss of Src-mediated AnxA2 phosphorylation and surface translocation; Reduced SEMA3D secretion and signaling	[138,152]
PDAC	Stroma signaling inhibitors in PDAC mouse models	Reduced PDAC metastasis	Suppression of AnxA2 phosphorylation	[139,140,153]
PDAC	Administration of AnxA2-targeting vaccine in PDAC mouse models	Improved survival	Inhibition of AnxA2-mediated oncogenic events	[155]
Bone marrow metastasis	AnxA2 depleted bone marrow cells in mice with prostate tumours	Reduced recruitment, growth, survival of prostate cancer cells in bone marrow	AnxA2 binding to CXCL12 and stromal-derived factor 1	[156]
Brain cancer	AnxA2 depletion in SK-N-BE(2) xenografts (pediatric neuroblastoma)	Improved chemotherapy	Attenuated NFκB activity	[157]
Brain cancer	AnxA2 depletion in U87 xenografts (glioblastoma multiforma, GBM)	Reduced GBM growth and improved survival	AnxA2-mediated STAT3 activation promotes EMT transition via miR155, oncostatin M receptor, cyclin D1	[158,159,160,161]
Breast cancer	Brain and lung metastasis of MDA-MB-231, MDA-MB-831, and MDA-MB-4175 after priming with AnxA2-depleted exosomes	Loss of favourable microenvironment decreased metastasis	Exosomal AnxA2 promotes tPA-dependent angiogenesis	[162]
Breast cancer	AnxA2 antibody conjugated and curcumin-loaded nanoparticles	Effective accumulation of curcumin in breast tumours	Improved targeted curcumin delivery	[163]
Breast and ovarian cancers	Administration of AnxA2 antibody conjugated to IgG or anticancer drugs to IGROV1 xenografts	Antibody-dependent cell toxicity and killing	Targeted delivery to tumours	[164,165,166]
TNBC	Administration of AnxA2 antibodies to TNBC xenografts	Inhibition of neoangiogenesis	Reduced AnxA2 phosphorylation and plasmin generation	[167,168,169]
Esophageal cancers	AnxA2 depletion in KYSE30 xenografts	Improved anti-cancer efficacy of Src and VEGF inhibitors	Loss of AnxA2 phosphorylation and Myc-Hif1α-VEGF activation	[170]
Non-small cell lung cancer (NSCLC)	AnxA2 depletion in cisplatin-resistant A549 xenografts	Increased cisplatin sensitivity	AnxA2-mediated JNK/c-Jun signaling reduces p53 activity	[171]
Lung cancer (NSCLC)	AnxA2 depletion in HCC827 and PC9 xenografts	ANXA2 depletion reversed EMT phenotype and gefitinib resistance	CAFs secrete HGF and IGF-1, which increases AnxA2 levels and phosphorylation, driving EMT	[172]
Acute lymphoblastic leukemia (ALL)	Administration of AnxA2/p11 inhibitor or AnxA2 antibodies to ALL mouse models	Reduced metastasis and improved chemotherapy	Blocked ability of AnxA2/p11 to promote adhesion, homing and engraftment of ALL cells	[173]
Brain cancer (Glioma)	Administration of AnxA2-antigen fusion peptides to GL261 tumour mouse models	Improved anti-tumour immunity	Monomeric AnxA2 binds to Toll-like receptor 2 to promote CD8^+^ T immune cell responses	[179]
Ovarian cancer	Administration of RNA nanoparticle with AnxA2 aptamer to SKOV3 xenografts	Improved doxorubicin delivery and efficacy	Targeting AnxA2 on ovarian cancer cells for drug delivery	[180]
Multiple myeloma (MM)	Administration of DNA aptamer targeting AnxA2 on ARP-1 xenografts	Blocked MM adhesion and proliferation	Targeting AnxA2 on cancer cell surface	[181]
Solid tumours	Administration of cyclic octapeptide targeting p-AnxA2 on 4T1 breast, HT1080 fibrosarcoma and BxPC-3 pancreatic tumours	Effective tumour imaging and monitoring if drug delivery	Elevated levels of phosphorylated AnxA2 in tumour tissues	[182]
Lung metastasis	Mouse lung colonization models (KRIB, B16F10, LLC). Pre-treatment with peptide targeting intracellular AnxA2	Reduced adhesion, migration and capacity to colonize lungs	Blocked interaction of AnxA2 with cytoskeleton	[183]
**(f) AnxA2 and Infection**				
**Disease**	**Model**	**Phenotype**	**Mechanism**	**Ref**
Bacteria-induced pulmonary inflammation	AnxA2 KO-mice	Increased susceptibility to bacterial infection	Toll-like receptor 4 (TLR4) trafficking defects causing enhanced TLR4 signaling	[184]
Fungal infection	AnxA2 KO-mice	Enhanced inflammatory response and death	Compromised fungal uptake, propagation and release	[185]
Macroautophagy	AnxA2 KO-mice	Reduced phagosome biogenesis and maturation in denditric cells	AnxA2 recruits phosphoinositides and PS to vesicular membranes, coordinating vesicular budding and homotypic fusion	[186]
Macroautophagy against bacterial infection	AnxA2 KO-mice	Severe lung injury, increased mortality; Elevated inflammatory cytokines, decreased bacterial clearance by macrophages, increased superoxide release in the lung	AnxA2 regulates autophagy and host immunity via Akt/mTOR/ULK signaling	[187]
Alzheimer’s disease	Alzheimer mouse models	AnxA2 is a therapeutic target in Alzheimer’s disease	AnxA2 binds phosphorylated preselenin-1 and VAMP8 to enable fusion of autophagosomes with lysosomes, which increases β-amyloid degradation	[188]
Acute febrile illness (anaplasmosis)	AnxA2 KO-mice	Increased susceptibility to *A. phagocytophilum* infection, causing splenomegaly, thrombo- and monocytopenia	AnxA2 binds sialostatin L2, which triggers pro-inflammatory cytokine production	[189]
Sepsis	AnxA2 KO-mice	Reduced bacterial clearance and survival	Increased IL-17 and reactive oxygen species production	[190]
Bladder infection	Administration of Ca^2+^ chelator or AnxA2/p11 inhibitor to mice infected with *E.coli*	Attenuated *E. coli* infection	Loss of AnxA2 as the receptor of bacterial protein YadC on bladder epithelial cells	[191]
*Rickettsia* infection	AnxA2 KO-mice	Blocked *Rickettsia* adherence to luminal side of blood vessels	Loss of AnxA2 as the receptor of *Rickettsia* on vascular endothelial cells	[193]
**(g) AnxA2 and Muscle Repair**				
**Disease**	**Model**	**Phenotype**	**Mechanism**	**Ref**
Dysferlinopathy	AnxA2 KO-mice	Poor sarcolemma repair, progressive and age-dependent decline in muscle function, lack of adipogenic replacement of myofibers	Similar to dysferlin deficiency but without chronic inflammation; AnxA2 target for dysferlinopathy	[194,195]
Limb girdle muscular dystrophy (LGMD) 2B	AnxA2 administration in mouse LGMD 2B model	Enhanced muscle loss		[195]
**(h) AnxA2 and Other Biological Activities**				
**Disease**	**Model**	**Phenotype**	**Mechanism**	**Ref**
Preeclampsia	AnxA2 KO-mice	Impaired embryo implantation and placentation	Impaired decidualization of endometrial stromal cells and uterine environment; AnxA2 acts as adhesion molecule during implantation	[196,197]
Chronic pain (tissue damage, harmful chemicals)	AnxA2 KO-mice	Enhanced TRPA1-dependent pain	AnxA2 is a ligand for TRPA1 responsible for ion channel regulation	[198]

Abbreviations: Akt, protein kinase B; ALL, acute lymphoblastic leukemia; ANO5, Anoctamin 5; Anx, annexin; ApoE, apolipoprotein E; COL6, collagen VI; CAFs, cancer associated fibroblasts; CVD, cardiovascular disease; EMT, epithelial-to-mesenchymal transition; FPR, formylated peptide receptor; GBM, glioblastoma multiforme; IgG, immunoglobulin G; I/R, ischemia/reperfusion; HFD, high-fat diet; Hif-1a, Hypoxia-inducible factor-1a; IL, interleukin; IPF, idiopathic pulmonary fibrosis; JNK, janus kinase; KLF2, kruppel-like factor 2; KO, knockout; LGMD, Limb girdle muscular dystrophy; LDLR, low density lipoprotein receptor; MAPK, mitogen-activated protein kinase; MI, myocardial infarction; miR, micro RNA; mTOR, mechanistic target of rapamycin; NFκB, nuclear factor kappa B; PCSK9, proprotein convertase subtilisin/kexin-9; PDAC, pancreatic ductal adenocarcinoma; PI3K, phosphoinositide-3-kinase; PKC, protein kinase C; PS, phosphatidylserine; SEMA3D, semaphoring 3D; SLE, systemic lupus erythematosus; SNARE, soluble NSF attachment protein receptor; SAT3, signal transducer and activator of transcription 3; STZ, streptozotocin; TFEB, transcription factor EB; TLR4, Toll-like receptor 4; TNBC, triple-negative breast cancer; tPA, tissue plasminogen activator; TRPA1, transient receptor potential ion channel A1; ULK, Unc-51 like autophagy activating *kinase*; VEGF, vascular endothelial growth factor; WT wildtype.

**Table 3:** Phenotypes observed in AnxA3 in vivo models.

Disease	Model	Phenotype	Mechanism	Ref
Ovarian Cancer	AnxA3 depletion in SKOV3 xenografts	Improved cisplatin sensitivity		[205]
Breast cancer	AnxA3 depletion in MDA-MB-231 xenografts	Reduced growth, angiogenesis and metastasis; Improved doxorubicin sensitivity	NFκB inhibition?	[200,206]
Pancreatic cancer	miR-382-mediated AnxA3 downregulation in BxPC-3 and PANC-1 xenografts	Reduced metastasis	PI3K/Akt pathway inhibition	[207]
Lung adenocarcinoma	AnxA3 depletion in A549 xenografts	Reduced xenograft growth and metastasis	Reduced MAPK signaling	[208]
Lung adenocarcinoma	AnxA3 depletion in CAFs; A549 xenografts	Increased cisplatin sensitivity	CAFs regulate AnxA3/JNK pathway that controls cisplatin-induced apoptosis	[209]
Hepatocellular carcinoma	HepG2, HuH7, sorafenib-resistant HepG2, patient-derived xenografts	Anti-AnxA3 mAb combined with sorafenib impaired tumour growth and reduced drug resistance	Attenuated PKCδ/p38-dependent apoptotic signaling confers drug resistance	[201]
Bone cancer-induced pain (BCP)	Adenoviral-mediated AnxA3 knockdown in metastatic lung BCP model	Microglial AnxA3 depletion alleviates BCP	Inhibited Hif-1α/VEGF signaling	[211]

Abbreviations: Akt, protein kinase B; Anx, annexin; BCP, bone cancer-induced pain; CAFs, cancer associated fibroblasts; Hif-1α, Hypoxia-inducible factor-1α; JNK, janus kinase; KO, knockout; MAPK, mitogen-activated protein kinase; miR, micro RNA; NFκB, nuclear factor kappa B; PI3K, phosphoinositide-3-kinase; PKC, protein kinase C; VEGF, vascular endothelial growth factor.

**Table 4:** Phenotypes observed in AnxA4 in vivo models.

Tissue/Disease	Model	Phenotype	Mechanism	Ref
Bladder	AnxA4 KO-mice	Normal urothelium	Barrier function? Membrane trafficking, bladder-voiding behavior?	[213,217]
Skin wound repair	Exogenous AnxA4 administration	Wound bleeding	Membrane repair?	[80]
Lung development	AnxA4-KO airway epithelial cells; Ex vivo cultured lungs	Impaired airway epithelial cell migration to distal airway tips	ERK1/2-dependent migration of airway epithelial progenitor cells	[218]
Cardiac function	AnxA4-KO cardiomyocytes	Enhanced contractility with β-AR agonists, elevated cAMP levels and response	Adenylyl cyclase 5 inhibition	[219,220]
Gallbladder cancer (GBC)	AnxA4 depletion in GBD-SD and -NOZ xenografts	Reduced tumour growth	Reduced oncogenic NFκB signaling	[221]
Breast cancer	AnxA4 depletion in MDA-MB-231 and MDA-MB-468 xenografts	Reduced tumour growth	Loss of AnxA4/AnxA1 interaction required for JNK/STAT3 signaling	[222]
Platinum resistance in endometrial carcinoma	AnxA4 overexpression in HEC1 xenografts	Increased cisplatin resistance	Increased activity of copper transporter ATP7A	[223]
Platinum resistance in ovarian cancer	AnxA4 depletion and AnxA4 mutant overexpression in RMG-I and NUGC3 xenografts	Increased cisplatin sensitivity	AnxA4 confers Ca^2+^- and chloride-dependent platinum resistance	[224]
Paclitaxel resistance in lung cancer	Peptide-mediated AnxA4 inhibition in A549 xenografts	Suppression of AnxA4-dependent paclitaxel resistance	FHIT-peptide delivery to inhibit AnxA4 membrane translocation	[225]

Abbreviations: Anx, annexin; β-AR, β-Adrenoreceptor; Ca^2+^, calcium; cAMP, cyclic adenosine monophosphate; ERK1/2, extracellular signal-regulated protein kinase 1/2; GBC, gallbladder carcinoma; JNK, Janus kinase; KO, knockout; NFκB, nuclear factor kappa B; STAT3, signal transducer and activator of transcription 3.

**Table 5:** Phenotypes observed in AnxA5 in vivo models.

**(a) AnxA5 and Development**				
**Tissue/Disease**	**Model**	**Phenotype**	**Mechanism**	**Ref**
Embryonal development	Targeted AnxA5-lacZ expression	Expression in perivascular cells of non-skeletal tissues	Angiogenesis differentiation? Endothelial cell maturation?	[234,235]
Bone and cartilage development	AnxA5 KO-mice; AnxA5/A6 Double-KO mice	Normal calcification during skeletal development		[236,238,239]
Bone cartilage development	AnxA5 KO-mice	Bone overgrowth at enthesis	AnxA5 prevents bone overgrowth triggered by mechanical forces	[240]
Hair bundle development	AnxA5 KO-mice	Normal stereocilia		[241]
**(b) Anxa5 and Anticoagulation**				
**Tissue/Disease**	**Model**	**Phenotype**	**Mechanism**	**Ref**
Placenta	Maternal AnxA5 KO-mice	Reduced litter size; Increased risk of foetal loss; Placental thrombosis	AnxA5-mediated anticoagulation via Ca^2+^-dependent binding and lattice formation on PS-rich membranes	[243,244,245]
Placenta	AnxA5 KO-mice	Zn^2+^ rescued anticoagulation and alleviated pregnancy loss in AnxA5-KOs	Alternative means for anticoagulation; Zn^2+^ elevates AnxA5 levels in WT mice	[246]
Thrombosis	Administration of echistatin-AnxA5 fusion protein to WT mice	Improved clotting and reduced wound healing time	Echistatin-annexin V fusion protein targets integrin α_IIb_β_3_ receptor and PS on platelets in thrombogenesis sites	[247]
Carotoid thrombosis	FeCl_3_-induced carotid thrombosis in WT mice	AnxA5-conjugated micelles for delivery of thrombolytic enzymes	AnxA5 binding to PS provides drug delivery for targeted thrombolysis	[248]
Thrombosis in abdominal sepsis	Administration of WT and mutant AnxA5 in septic animals	AnxA5 inhibits thrombin formation	Ca^2+^-dependent binding of WT, but not mutant AnxA5, to PS	[249]
Thrombus formation after blood vessel injury	Administration of AnxA5 dimer in laser-induced injury and tail tip bleeding model	AnxA5 inhibits thrombin formation and clot formation	Ca^2+^-dependent binding of AnxA5 dimer to PS-exposing platelets	[252]
**(c) AnxA5 and Wound Repair, Inflammation**				
**Disease**	**Model**	**Phenotype**	**Mechanism**	**Ref**
Skin wound repair	AnxA5 KO-mice; Exogenous AnxA5 administration	Skin wound repair unaltered	AnxA5 binding to PS? Membrane repair?	[80]
Lung fibrosis and inflammation	Aerosol delivery of AnxA5	Alveolar AnxA5 promotes lung inflammation and fibrosis	AnxA5 binding to PS and signaling?	[251]
Muscle dystrophy	Myofibers from ANO5 KO-mice	Loss of Anx-dependent cap formation at injury site	Diminished AnxA5 at injury site	[287]
**(d) AnxA5 and Cardiovascular Disease**				
**Disease**	**Model**	**Phenotype**	**Mechanism**	**Ref**
Diagnosis/detection of atherosclerotique plaque	HFD-fed ApoE KO-mice; Other CVD models	Radio-, fluorescently labeled AnxA5 or AnxA5-conjugated gold particles to detect plaque	AnxA5 binding to PS on apoptotic cells	[226,253,254,255,256]
Detection of cell death in stroke	Ethanol-induced cell death in femur muscle vs. stroke in mice	Dual-labeled AnxA5 (^99m^TC, Alexa) to detect apoptosis	Dual-labeled AnxA5 failed to recognize cell death in stroke	[272]
CVD treatment	AnxA5 administration in HFD-fed ApoE KO- mice	Reduced plaque inflammation and plaque size	AnxA5 binding to PS on apoptotic cells reduced monocyte recruitment	[256,257]
Myocardial infarction (MI)	AnxA5 treatment of hypercholesterolemic ApoE3 Leiden mice	Reduced plaque inflammation and plaque size	AnxA5 binding to PS on apoptotic cells	[258]
MI	SDF-1-AnxA5 fusion protein treatment in MI mouse model	Cardioprotection	AnxA5 binding to PS on apoptotic cells	[259]
Nonalcoholic steatohepatitis (NASH)	AnxA5 administration in HFD-induced NASH mouse model	AnxA5 improves steatosis, inflammation and fibrosis	AnxA5 binding to PKM2 in macrophages enables switch from glycolysis to oxidative phosphorylation	[260]
**(e) AnxA5 and Cancer**				
**Disease**	**Model**	**Phenotype**	**Mechanism**	**Ref**
Hepatocellular carcinoma	AnxA5 overexpression in Hca-P xenografts	Increased growth and metastasis	MAPK and Rac1 signaling	[261,262]
Glioblastoma	Intracranial glioma mouse model: implanted U97 cells ± AnxA5 depletion/overexpression	AnxA5 promotes glioma tumourgenicity	PI3K/Akt/NFκB signaling?	[263]
Renal cell carcinoma	AnxA5 depleted Caki-1 xenografts	Reduced tumourgenicity	Reduced PI3K/Akt/mTORC1 activity and MMP expression	[264]
Gefitinib resistance in non-small cell lung cancer	AnxA5 knockdown in gefitinib-resistant PC9 xenografts	Restoration of gefitinib sensitivity	G2/M cell cycle arrest, inhibition of apoptosis	[265]
Melanoma	AnxA5 administration in B16F10 xenografts	Reduced tumour size; Increased tumour necrosis	Reduced VEGF expression and angiogenesis	[266]
Breast cancer immunotherapy	Intra-tumoural AnxA5 release in 4T1 breast tumours	AnxA5 triggers anti-tumour immune response that leads to tumour regression	AnxA5 binds PS on apoptotic cells to block phagocytic clearance, followed by stimulation of immune system	[267]
Lung cancer immunotherapy	AnxA5 administration following cisplatin treatment in TC-1 tumour model	AnxA5 increases immune response and alleviates cisplatin-mediated immunosuppression	AnxA5 can deliver antigenic peptides to the tumour environment	[268,269,270]
Ovarian cancer prodrug and imunotherapy	Targeted delivery of mCTH-AnxA5 fusion protein together with immunostimulants to ID8 tumours	AnxA5 targets mCTH to tumour to convert nontoxic to toxic drug, increased cell death improves immune-directed tumour destruction	AnxA5 binding to PS on tumour and tumour vasculature as vehicle to enable enzyme-prodrug approach	[271]
Detection of cancer cell death after chemotherapy	Ganciclovir-treated NG4TL4 sarcoma xenografts expressing herpes simplex virus-TK	^111^In- and Dota-peptide-labeled AnxA5 monitors drug efficacy	AnxA5 binding to PS on apoptotic tumour cells	[273]
Detection of cancer cell death after chemotherapy	Etoposide- and cyclophosphamide-treated EL-4 xenografts	AnxA5-conjugated ultrasmall super-paramagnetic iron oxide detects cancer cell apoptosis	AnxA5 binding to PS on apoptotic tumour cells	[274]
Detection of cancer cell death after chemotherapy	Cisplatin-treated MDA-MB-231 xenografts	AnxA5-conjugated magnetic beads	AnxA5 binding to PS on apoptotic tumour cells assessed by ultrasound	[275]
**(f) AnxA5 and Detection of EVs and Apoptosis**				
**Tissue/Disease**	**Model**	**Phenotype**	**Mechanism**	**Ref**
Detection of extracellular vesicles (EVs)	Human HCC827 xenografts	AnxA5-conjugated magnetic beads	AnxA5 binding to PS enables EV isolation	[276]
Detection of liver apoptosis and cancer	Anti-Fas antibody model for fast hepatic apoptosis; Burkitt’s lymphoma model	^68^Ga-labeled AnxA5; ^99m^Tc-labeled AnxA5	AnxA5 binding to PS on apoptotic cells	[277,278]
Vascular apoptosis	Ethanol-induced corneal apoptosis in mice	AnxA5 fused to *Renilla* luciferase mutant as biosensor	AnxA5 binding to PS on apoptotic cells	[279]
Efferocytosis	Injection of Jurkat cells labeled with pH-sensitive AnxA5 probe into peritoneal cavity of mice	Ex vivo measurement of phagocytosis of apoptotic cells by peritoneal macrophages	AnxA5 binding to PS and exposure to different pH environments	[280]
Immunogenicity of apoptotic cells	Induction of necrosis by mechanical stress in AnxA5-KO mice	AnxA5 is required for immune response against dead cells	AnxA5 binding to PS on apoptotic cells	[284,285]
Immune tolerance	Dectin-1 KO-mice	Autoimmune pathologies (autoantibodies, splenomegaly, stronger immune response)	Dectin-1 on dendritic cells binds to core domain of annexins (incl. AnxA5) for uptake of apoptotic cells to establish tolerance for self-antigens	[286]

Abbreviations: Akt, protein kinase B; ANO5, anoctamin-5; Anx, annexin; apoE, apolipoprotein E; Ca^2+^, calcium; CVD, cardiovascular disease; EVs, extracellular vesicles; HFD, high-fat diet; KO, knockout; mCTH, mutated cystathionine gamma-lyase; MI, myocardial infarction; MMP, matrix metalloproteases; mTORC1, mammalian target of rapamycin complex 1; NASH, nonalcoholic steatohepatitis; PKM2, pyruvate kinase 2; PS, phosphatidylserine; SDF-1, stromal cell-derived factor 1; STAT3, signal transducer and activator of transcription 3; TK, thymidine kinase; VEGF, vascular endothelial growth factor; WT, wildtype.

**Table 6:** Phenotypes observed in AnxA6 in vivo models.

**(a) AnxA6 and Cardiac Function**				
**Tissue/Disease**	**Model**	**Phenotype**	**Mechanism**	**Ref**
Cardiomyopathy Heart failure	Heart-specific AnxA6 WT/mutant overexpression	Amplitude Ca^2+^ transients; Ca^2+^ flux and signaling; Contractility	Ca^2+^-dependent ion pumps/channels in cardiomyocytes	[306,308]
Cardiac function	AnxA6-KO cardiomyocytes	Ca^2+^ flux and signaling; Contractility	Ca^2+^-dependent ion pumps/channels	[307]
Cardiac function	AnxA6 KO-mice	Normal cardiac function	Redundancy?	[312]
**(b) AnxA6 and Skeletal Development**				
**Tissue/Disease**	**Model**	**Phenotype**	**Mechanism**	**Ref**
Bone and cartilage development	AnxA6 KO-mice; AnxA5/A6 Double-KO mice	Normal skeletal development	Redundancy?	[238,239]
Bone and cartilage development	AnxA6 KO-mice, Newborns	Reduced mineralization of growth plate cartilage; Delayed chondrocyte differentiation	Loss of AnxA6-dependent PKCα translocation and signaling	[316]
Osteoarthritis	AnxA6 KO-mice	Reduced knee cartilage destruction	Loss of AnxA6/p65 (NFκB) interaction	[317,318]
Pain in osteoarthritis	AnxA6 KO-mice	Increased sensitivity to mechanical/chronic pain stimuli	Increased cation channel Piezo2 activity in sensory neurons	[320]
**(c) AnxA6 and Immune Response**				
**Tissue/Disease**	**Model**	**Phenotype**	**Mechanism**	**Ref**
Immune system	AnxA6 KO-mice	Normal B and T cell development	Redundancy?	[314]
Immune response	AnxA6 KO-mice	Reduced CD4^+^ T lymphocyte activation	Membrane organisation; IL-2 signaling	[321]
**(d) AnxA6 and Homeostasis**				
**Tissue/Disease**	**Model**	**Phenotype**	**Mechanism**	**Ref**
Membrane order	AnxA6-KO MEFs	Microdomain organisation	Membrane domain/lipid distribution	[319]
Ca^2+^ homeostasis	AnxA6-KO (liver, retina) AnxA6-KO MEFs	Dysfunctional mitochondria	Loss of AnxA6/Drp1 interaction	[289]
**(e) AnxA6 and Lipid/Glucose Homeostasis**				
**Tissue/Disease**	**Model**	**Phenotype**	**Mechanism**	**Ref**
Adipose tissue	AnxA6 KO-mice	Reduced WAT weight gain after HFD; Increased adiponectin secretion	Triglyceride metabolism; Lipid droplet functions; Adipokine secretion	[324,325,326]
Liver regeneration	AnxA6 KO-mice	Hypoglycemia; De-regulated hepatic gluconeogenesis	Loss of alanine uptake and SNAT4 transporter cell surface localization	[329]
HFD-induced fatty liver and insulin resistance	AnxA6 KO-mice	Altered insulin signaling; Increased glycogen storage; Failure to downregulate hepatic gluconeogenesis	Loss of AnxA6 scaffold and membrane transport functions	[326]
Neuronal damage, motor coordination	AnxA6 KO-mice; NPC1 KO-mice; AnxA6/NPC1 Double-KO mice	Normal in AnxA6-KO; Cerebellar degeneration similar in NPC1-KO and AnxA6/NPC1 Double-KO		[339]
Liver dysfunction	AnxA6 KO-mice; NPC1 KO-mice; AnxA6/NPC1 Double-KO mice	AnxA6/NPC1 Double-KO compared to NPC1-KO: Increased liver inflammation; Altered mitochondrial morphology; Higher turnover of neutral lipids and glycogen; Reduced lifespan	Loss of AnxA6 scaffold to control cholesterol homeostasis, inter-organelle communication and macrophage infiltration	[340]
**(f) AnxA6 and Cancer**				
**Disease**	**Model**	**Phenotype**	**Mechanism**	**Ref**
EGFR-related cancer	AnxA6 overexpression in A431 xenografts	Reduced tumour growth; Impact on EGFR-targeted drug efficacy	AnxA6-mediated and PKCα- and p120GAP- dependent inhibition of EGFR/Ras/MAPK signaling	[345,346]
Triple-negative breast cancer (TNBC)	AnxA6 depletion in BT-549 xenografts	Early onset and rapid tumour growth; Impact on EGFR-targeted drug efficacy	AnxA6- and RASGRF2- dependent EGFR/Ras/MAPK signaling	[347,348,349]
Drug resistance in breast cancer	MMTV-PyMT and 4T1 breast cancer models	Chemotherapy-elicited secretion of AnxA6-containing EVs from tumours with enhanced metastatic capacity	Pulmonary NFκB-dependent endothelial activation, elevated cytokine secretion	[351]
Pancreatic ductal adenocarcinoma (PDAC)	PDAC mouse model	ANXA6-depleted EVs from CAFs impaired PDAC and metastasis	CAF-cancer cell communication via ANXA6/LRP1/TSP1	[352]
Gastric cancer	NUGC3 xenografts	AnxA6-containing EVs from CAFs contribute to drug resistance	AnxA6-mediated activation of integrin β1, FAK and YAP1	[353]
**(g) AnxA6 and Membrane Repair**				
**Disease**	**Model**	**Phenotype**	**Mechanism**	**Ref**
Muscle dystrophy	AnxA6-KO in zebrafish	Impaired membrane repair	Cooperation with dysferlin	[293]
Muscle dystrophy	Mouse models with Dysferlin mutations	Impaired AnxA6 recruitment for membrane repair	Cooperation with dysferlin and other annexins to seal injured membrane	[354,355,356]
Muscle dystrophy	Mouse models for muscular dystrophy	AnxA6 is a modifier gene required for membrane repair after muscle injury	AnxA6 mutants contribute to defects in sarcolemma resealing	[295]
Muscle dystrophy	Mouse models for muscular dystrophy	Delivery of AnxA6 promotes membrane repair in muscle	AnxA6 upregulation or administration of recombinant AnxA6 improves repair	[357,358]
Muscle dystrophy	Myofibers from ANO5 KO-mice	Loss of Anx-dependent cap formation at injury site	Lack of AnxA6 accumulation at injury site	[287]

Abbreviations: ANO5, anoctamin-5; Anx, annexin; Ca^2+^, calcium; CAFs, cancer associated fibroblasts; Drp1, dynamin-related protein 1; EGFR, epidermal growth factor receptor; EVs, extracellular vesicles; FAK, focal adhesion kinase; HFD, high-fat diet; IL, interleukin; KO, knockout; LRP1, low density lipoprotein receptor related protein 1; MAPK, mitogen-activated protein kinase; MMTV-PyMT, mouse mammary tumor virus-polyoma middle tumor-antigen; NFκB, nuclear factor kappa B; p120GAP, p120 GTPase activating protein; PDAC, pancreatic ductal adenocarcinoma; PKCα, protein kinase Cα; RASGRF2, Ras protein specific guanine nucleotide exchange factor; SNAT4, sodium-coupled neutral amino acid transporter 4; TNBC, triple negative breast cancer; TSP1, thrombospondin 1; WAT, white adipose tissue. YAP1, yes-associated protein 1.

**Table 7:** Phenotypes observed in AnxA7 in vivo models.

**(a) AnxA7 and Various Disease Phenotypes**				
**Tissue/Disease**	**Model**	**Phenotype**	**Mechanism**	**Ref**
Development	AnxA7 KO-mice ^1^ (*neo* cassette in intron 5-exon 6)	Embryonal lethality due to cerebral haemorrhage	Vascular development?	[361]
Pancreas	Heterozygous AnxA7 KO-mice ^2^ (*neo* cassette in intron 5-exon 6)	Islet hyperplasia; β-cell hypertrophy; Defects in Ca^2+^ homeostasis and insulin secretion	Reduced InsP_3_ receptor levels; Defective InsP_3_ receptor and RyR-mediated Ca^2+^ release	[361,370]
Pancreas	AnxA7 KO-mice ^3^ (*neo* cassette in exon 8)	Normal pancreatic β-cells and insulin secretion	Normal Ca^2+^ homeostasis	[371]
Heart	AnxA7 KO-mice ^3^	De-regulated cardiomyocyte contraction; Heart arrhythmia; Cardiac remodelling	Defects in Ca^2+^ homeostasis; Impaired integrity of basement cardiomyocyte membrane; Lack of collagen; Ca^2+^-dependent NFAT activity	[371,372,374]
Eryptosis	AnxA7 KO-mice ^3^	Erythrocyte resistance to osmotic shock; Protection against *plasmodium* infection; Increased adhesion to endothelial cells	Elevated COX-dependent PGE_2_ production, Ca^2+^ homeostasis and PS exposure	[375,376,377,379]
Uterus	AnxA7 KO-mice ^3^	Endometrial receptivity and implantation	Elevated COX-dependent PGE_2_ production	[380]
Intestine	AnxA7 KO-mice ^3^	Compromised glucocorticoid-induced gastric acid secretion	Elevated COX-dependent PGE_2_ production	[378]
Liver	AnxA7 KO-mice ^3^	Decreased glucose tolerance Elevated plasma prostaglandins	Elevated hepatic COX activity	[366]
Brain	AnxA7 KO-mice ^3^	Astrocyte proliferation	Ca^2+^ homeostasis	[381]
Brain injury	Localized AnxA7 depletion in intracerebral- or subarachnoid hemorrhage- induced brain injury (rats, mice)	Induction of neuronal apoptosis	Pre-synaptic glutamate release and post-synaptic NMDA receptor trafficking	[382,383]
Atherosclerosis	HFD-fed ApoE KO-mice	ABO treatment to target AnxA7-GTPase activity; Reduced plaque size; Increased plaque stability	Suppression of PC-specific PLC in endothelial cells	[386,387,388]
**(b) AnxA7 and Cancer**				
Cancer	Cancer-prone phenotype of heterozygous AnxA7 KO-mice ^2^	Genomic instability affecting tumour suppressor, DNA-repair, apoptosis-related genes	Tumour suppressor activity? Loss of AnxA7 GTPase activity?	[389]
Adeno-carcinoma	A549 xenografts in avian embryo model	SEC treatment to increase AnxA7 GTPase activity	Nuclear translocation of integrin β4 reduces tumour growth	[390]
Prostate cancer	PC3 xenografts	SEC treatment to increase AnxA7 GTPase activity	Inhibition of mTORC1/STAT3 signaling	[391]
Hepatocellular carcinoma (HCC)	AnxA7 overexpressing HCC xenografts	Decreased tumourgenicity and metastasis	Reduced VEGF levels	[392]
HCC	HCC xenografts	Decreased tumourgenicity and metastasis	miR-124-3p mediated AnxA7 downregulation	[393]
Gastric cancer	AnxA7 depletion in BGC823 xenografts	Decreased tumour growth	Increased apoptosis	[394]

Abbreviations: ABO, 6-amino-2, 3-dihydro-3-hydroxymethyl-1, 4-benzoxazine; Akt, protein kinase B; Anx, annexin; apoE, apolipoprotein E; Ca^2+^, calcium; COX, cyclooxygenase; HCC, hepatocellular carcinoma; HFD, high-fat diet; InsP_3_, inositol 1,4,5-trisphosphate; KO, knockout; miR, micro RNA; mTORC1, mammalian target of rapamycin complex 1; NFAT, nuclear factor of activated T cells; NMDA, N-methyl-D-aspartate; PGE_2_, prostaglandin E_2_; PLC, phospholipase C; PC, phosphatidylcholine; PS, phosphatidylserine; RyR, ryanodine receptor; SEC, (S)-ethyl 1-(3-(4-chlorophenoxy)-2-hydroxypropyl)-3- (4 methoxyphenyl)-1H-pyrazole-5-carboxylate; STAT3, signal transducer and activator of transcription 3; VEGF, vascular endothelial growth factor. ^1^ non-viable AnxA7 knockout [355]. ^2^ viable heterozygous AnxA7 knockout [355,383]. ^3^ viable AnxA7 knockout [365].

**Table 8:** Phenotypes observed in AnxA8 in vivo models.

Tissue/Disease	Model	Phenotype	Mechanism	Ref
Preimplantation period in pregnancy	Intra-uterine injection of AnxA8-depleted endometrial cells in mice	Reduced implantation sites in the uterine horn	AnxA8 promotes endometrial cell proliferation via Akt signaling	[400]
Leukocyte recruitment to inflammatory-activated endothelial cells	AnxA8-KO	Impaired leukocyte rolling/adhesion to inflammatory-activated postcapillary venules	AnxA8-dependent cell surface delivery of CD63	[402]

Abbreviations: Akt, protein kinase B; Anx, annexin; KO, knockout.

**Table 9:** Phenotypes observed in AnxA10 in vivo models.

Disease	Model	Phenotype	Mechanism	Ref
Neuropathic pain	AnxA10 depletion in spinal nerve ligation (SNL) in mice	Attenuated SNL-induced neuropathic pain (hyperalgesia)	AnxA10 in neuronal cell and astrocytes	[413]
Neuropathicpain	AnxA10 depletion in spinal nerve ligation (SNL) in rats	Attenuated SNL-induced neuropathic pain (hyperalgesia)	Blocked activation of NFκB, TNFα, IL1β, metalloproteases	[414]

Abbreviations: Anx, annexin; IL1β, interleukin 1β; KO, knockout; NFκB, nuclear factor kappa B; TNFα, tumor necrosis factor α; SNL, spinal nerve ligation.

**Table 10:** Phenotypes observed in AnxA11 in vivo models.

Disease	Model	Phenotype	Mechanism	Ref
Hepatocellular carcinoma (HCC)	AnxA11 up-/downregulation in HCC xenografts and pulmonary metastatic tumours	AnxA11 levels modulate tumour growth and progression	miR-16-5p dependent AnxA11 levels determine Akt activity	[421]
Hepatocellular carcinoma (HCC)	AnxA11 depletion in Hca-P xenografts	Increased tumour growth and lymph node metastasis, 5-FU chemoresistance	Increased c-JUN activity	[422]
Bevacizumab efficacy in metastatic colorectal cancer	AnxA11 R230C variant (rs1049550) in SW480 xenografts	Improved suppression of tumour growth and progression		[424]

Abbreviations: 5-fluorouracil, 5-FU; Akt, protein kinase B; Anx, annexin; HCC, hepatocellular carcinoma; KO, knockout; miR, micro RNA.

## Abbreviations

ABO: 6-amino-2, 3-dihydro-3-hydroxymethyl-1, 4-benzoxazine; Akt, protein kinase B; ALS, amyotrophic lateral sclerosis; Anx, annexin; APL, acute promyelocytic leukemia; apoE, apolipoprotein E; β-AR, β-adrenoreceptor; Ca^2+^, calcium; CAFs, cancer associated fibroblasts; COL6, collagen VI; COX, cyclooxygenase; cPLA2, cytoplasmic phospholipase A2; EGFR, epidermal growth factor receptor; EMT, epithelial-to-mesenchymal transition; EVs, extracellular vesicles; FPR, formylated peptide receptor; GBM, glioblastoma multiforma; HFD, high-fat diet; IL, interleukin; KO, knockout; LDLR, low density lipoprotein receptor; MAPK, mitogen-activated protein kinase; MI, myocardial infarction; MVBs, multivesicular bodies; NFκB, nuclear factor kappa B; PHx, partial hepatectomy; PCSK9, proprotein convertase subtilisin/kexin-9; PDAC, pancreatic ductal adenocarcinoma; PGE2, prostaglandin E2; PI3K, phosphoinositide-3-kinase; PKC, protein kinase C; PS, phosphatidylserine; SEMA3D, semaphorin 3D; RyR, ryanodine receptor; SLE, systemic lupus erythematosus; SNARE, soluble NSF attachment protein receptor; SNAP23, synaptosomal-associated protein 23; SNL, spinal nerve ligation; SNP, single nucleotide polymorphism; STAT3, signal transducer and activator of transcription 3; STZ, streptozotocin; TLR4, Toll-like receptor 4; tPA, tissue plasminogen activator; TNBC, triple-negative breast cancer; TRPA1, transient receptor potential ion channel A1; VAMP2, vesicle-associated membrane protein 2; VEGF, vascular endothelial growth factor; WAT, white adipose tissue.
